# Neopapillarine, an Unusual Coumarino-Alkaloid from the Root Extract of *Neocryptodiscus papillaris* with Cytotoxic Activity on Renal Cancer Cells

**DOI:** 10.3390/molecules25133040

**Published:** 2020-07-03

**Authors:** Fatma Tosun, Feyyaz Mıhoğlugil, John A. Beutler, Esra Eroğlu Özkan, Mahmut Miski

**Affiliations:** 1 School of Pharmacy, Department of Pharmacognosy, Istanbul Medipol University, 34810 Istanbul, Turkey; 2 Faculty of Pharmacy, Department of Pharmacognosy, Cyprus International University, 99258 Nicosia, Northern Cyprus; fmihoglugil@ciu.edu.tr; 3 Molecular Targets Program, Center for Cancer Research, National Cancer Institute, Frederick, MD 21702, USA; beutlerj@mail.nih.gov (J.A.B.); eseroglu@istanbul.edu.tr (E.E.Ö.); 4 Faculty of Pharmacy, Department of Pharmacognosy, Istanbul University, 34116 Istanbul, Turkey

**Keywords:** *neocryptodiscus papillaris*, apiaceae, simple coumarins, furanocoumarins, prenylated coumarins, coumarino-alkaloid, cytotoxic activity

## Abstract

Several simple and prenylated coumarin derivatives were isolated from the dichloromethane extract of the root of *Neocryptodiscus papillaris* based on moderate cytotoxic activity of the extract in COLO205, KM12 and MCF7 cancer cells. While the major prenylated furanocoumarin derivatives and osthol isolated from the dichloromethane extract were responsible for the activity in the colon and breast cancer cell lines, the 4′-acylated osthol derivatives including a novel coumarino-alkaloid; neopapillarine) demonstrated selective cytotoxic activity in A498 and UO31 renal cancer cell lines.

## 1. Introduction

The flora of Turkey is highly diverse, with about seed plant species, 3100 of which are endemic [1]. As part of a long-term collaboration to explore the phytochemistry of rare, endemic, and medicinal Turkish plants, we evaluated the anticancer properties of extracts and pure compounds using cancer cell growth inhibition assays as well as molecularly targeted assays. We have focused on the family Apiaceae, a notably diverse plant family in Turkey, with 100 genera, e.g., [2]. Over 140 extracts from over 40 species of Turkish Apiaceae were tested by both cell growth and biochemical assays at the Molecular Targets Program, National Cancer Institute (NCI). *Neocryptodiscus* [3] is a small genus of the family Apiaceae represented by five species worldwide [4], which have not been investigated for their non-volatile secondary metabolites. *Neocryptodiscus papillaris* (Boiss.) Herrnst. & Heyn (*syn. Prangos papillaris* (Boiss.) Menemen) is a rare species only found in a small area of the Northern Mesopotamia region [5].

## 2. Results

**Figure 1:**
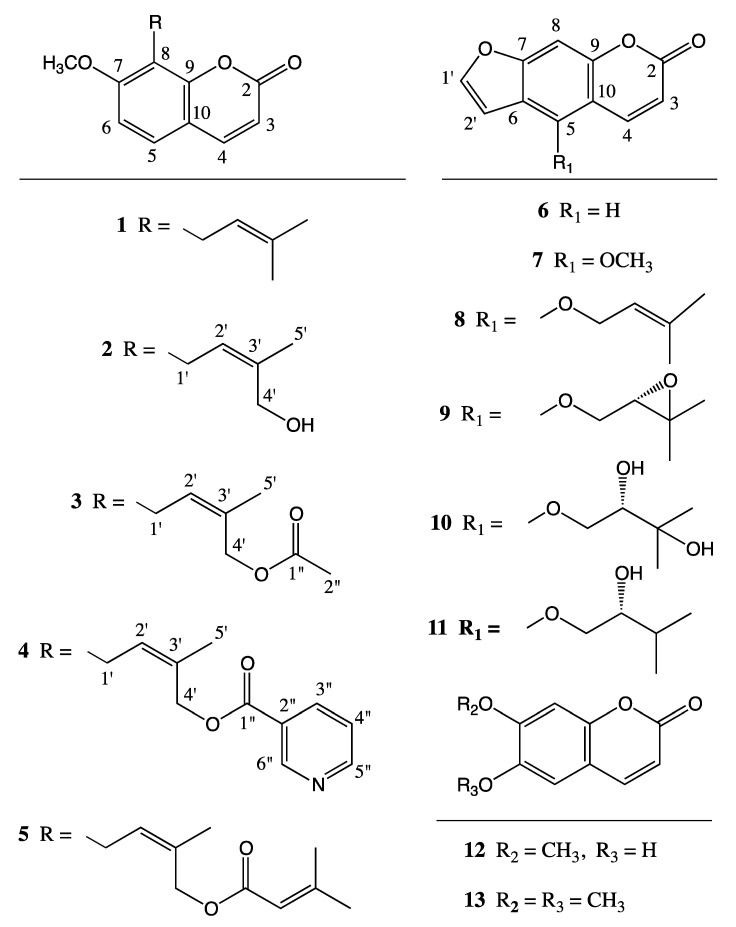
Structures of the coumarin derivatives isolated from the roots of *Neocryptodiscus papillaris*.

Cytotoxicity testing of the root and fruit extracts of *N. papillaris* showed moderate activity against COLO205, KM12, MCF7, A498 and UO31 cancer cell lines, with the dichloromethane root extract the being most active [6]. In addition to the various known coumarin derivatives, e.g., osthol (**1**) [7], 4′-senecioyloxyosthol (**5**) [8], psoralen (**6**), bergapten (**7**), isoimperatorin (**8**) [9], oxypeucedanin (**9**) [10], oxypeucedanin hydrate (**10**) [10,11], pranferol (**11**) [12], scopoletin (**12**), and scoparone (**13**) [13]; the fractionation of the dichloromethane extract of the root of *N. papillaris* yielded several novel osthol derivatives, i.e., 4′-hydroxyosthol (**2**), 4′-acetoxyosthol (**3**) and neopapillarine (**4**) (Figure 1).

In addition to their cytotoxic and anticancer activities [14,15], natural and semi-synthetic coumarins possess many biological activities properties [16] such as antiviral [17,18,19], antifungal, antioxidative [20,21], antibacterial [22] and anti-inflammatory activities [23]. It should be noted that aflatoxins, potent hepatotoxic, carcinogenic, mutagenic and teratogenic mycotoxins isolated from *Aspergillus* species, also contain a coumarin nucleus as part of their polycylic heterocyclic structure [24].

**Figure 2:**
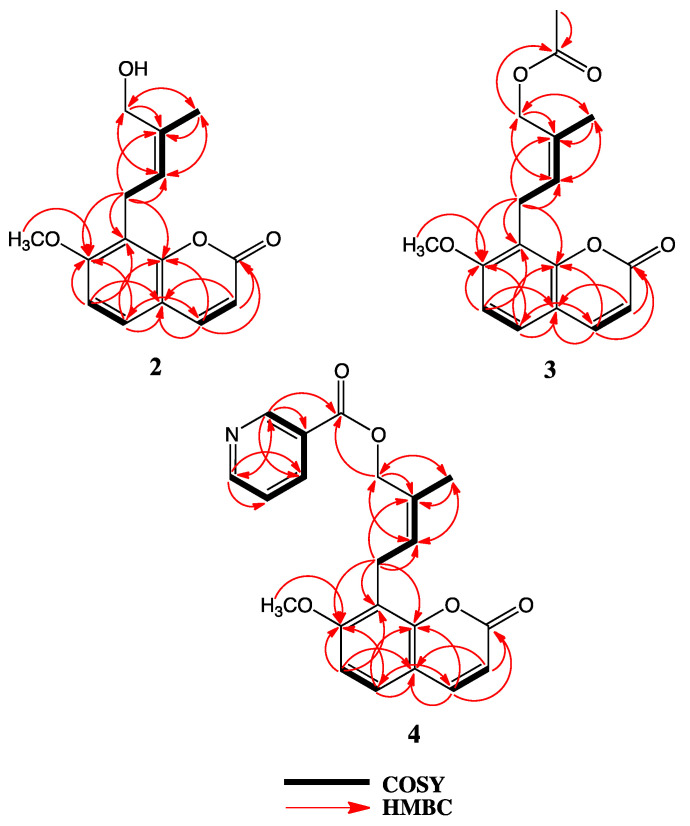
2D-COSY and HMBC interactions of compounds **2**, **3** and **4**.

**Figure 3:**
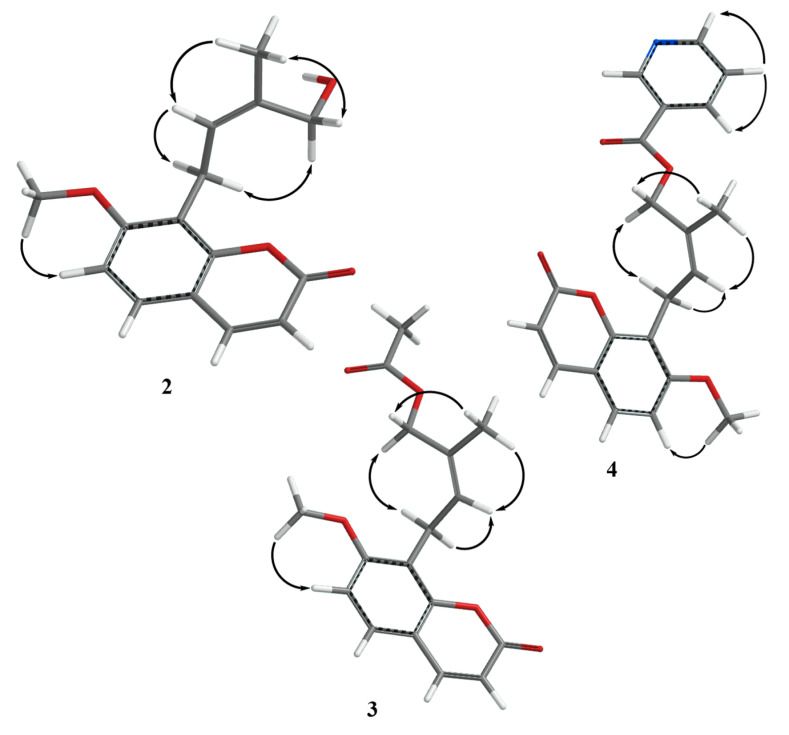
NOE interactions observed in the 2D-NOESY spectra of compounds **2**, **3** and **4** [27].

The ^1^H-NMR spectrum of 4′-hydroxyosthol (**2**) (Table 1) was similar to that of osthol (**1**) with the exception of the lack of one of the vinylic methyl group signals. The presence of a methylene singlet at δ_H_ 4.41 ppm (2H) suggested that the second vinylic methyl group of osthol was hydroxylated. The HRESIMS data of **2** showed a protonated molecular peak at *m/z* 261.1115 [M + H]^+^ (calcd. 261.1121, err. 2.55 ppm) indicating a molecular formula of C_15_H_16_O_4_ for **2** which that is in agreement with the hydoxylated isoprenyl side chain added to the osthol structure. Although this compound has previously been reported as a rat metabolite of osthol [25], both the ^1^H-NMR and ^13^C–NMR spectra of the metabolite were reported in deuterated dimethylsulfoxide and the reported chemical shifts do not closely match the ^1^H-NMR and ^13^C-NMR spectra of **2** recorded in CDCl_3_. Thus, in order to confirm the correct hydroxyosthol structure for **2**, the ^13^C-NMR as well as the COSY, NOESY, HSQC and HMBC spectra of **2** were recorded in CDCl_3_ (see Table 1, Figure 2, Figure 3 and Supplementary Materials). The NOESY spectrum of **2** exhibited interactions between the C-5′ methyl protons and H-2′ as well as H-4′ (Figure 3) suggesting the presence of a hydroxyl group on the osthol C-4′ methyl. Furthermore, 2D-COSY, HSQC and HMBC data (see Figure 2, Figure 3 and Supplementary Materials information) confirmed the structure of **2** as 4′-hydroxyosthol.

The ^1^H-NMR spectrum of 4′-acetoxyosthol (**3**) (Table 1) clearly suggested that **3** is the acetylated derivative of **2**. The presence of an acetyl methyl group signal in the ^1^H-NMR spectrum at δ_H_ 2.09 ppm (3H, s) and 0.44 ppm downfield shift of the C-4′ methylene protons [i.e., at δ_H_ 4.85 ppm (br s, 2H)] strongly suggested that the C-4′ hydroxymethylene group was esterified with an acetyl group in **3**. In addition, the position of the acetoxy group was corroborated by the 2D NOESY spectrum as C-4′ and furthermore, a sodium adduct molecular peak observed at *m/z* 325.1039 [M + Na]^+^ (calcd. 325.1052, err. 4.03 ppm) in the HRESIMS spectrum of **3** confirmed the structure of **3** as 4′-acetoxyosthol.

The ^1^H-NMR spectrum of neopapillarine (**4**) (Table 1) showed similar signals to that those of **3**. However, instead of an acetyl methyl group observed at δ_H_ 2.09 ppm in the ^1^H-NMR spectrum of **3**, the ^1^H-NMR spectrum of **4** contained four proton signals in the aromatic region of the spectrum. The chemical shifts and multiplicities of these four protons were almost identical to those of nicotinic acid esters [26], thus, the acid portion of the ester group in **4** should be nicotinic acid. The 2D NOESY spectrum of **4** indicated that the nicotinoyloxy group was located on the C-4′ methyl group (Figure 3) as with the acetoxy group of **3**. In addition, the HRESIMS spectrum of **4** displayed a protonated molecular peak at *m/z* 366.1337 [M + H]^+^ (calcd. 366.1341, err. 1.23 ppm) confirming a molecular formula of C_21_H_19_NO_5_ for **4** which that is in agreement with a 4′-nicotinoyloxyosthol structure for compound **4**.

The cytotoxic activity of the coumarins isolated from *N. papillaris* was tested in A498 and UO31 renal cancer cell lines (Table 2). While most furanocoumarins and simple osthol derivatives isolated from the root of *N. papillaris* showed no or weak inhibitory activity against these cell lines, osthol derivatives that contain larger 4′-acyloxy groups such as senecioyloxy (i.e., **5**) or nicotinoyloxy (i.e., **4**) group displayed a moderate inhibitory activity against the UO31 cell line. Furthermore neopapillarine (**4**), the 4′-nicotinoyloxy derivative of osthol, exhibited better inhibitory activity in the A498 renal cancer cell line in comparison with the 4′-senecioyloxy derivative of osthol (**5**).

## 3. Discussion

The Investigation of the dichloromethane extract of the root of *N. papillaris*, a rare and endemic Apiaceae plant, yielded several coumarin derivatives with cytotoxic activity including a novel coumarino-alkaloid compound; named neopapillarine (**4**). The prenylated major coumarins of the root extract were osthol (**1**), isoimperatorin (**8**) and oxypeucedanin (**9**). Osthol (**1**) showed 20 and 24 μM IC_50_ values in COLO205 colon cancer and MCF7 breast cancer cell lines, respectively. Oxypeucedanin (**9**) displayed 30 and 31 μM IC_50_ values in the COLO205 and KM12 colon cancer cell lines, but much weaker IC_50_ values (i.e., ca. 100 μM, 720 μM and 630 μM) against the MCF7 mammalian and A498 and UO31 renal cancer cell lines, respectively. Isoimperatorin (**8**) showed an IC_50_ value of 14 μM in the COLO205 cancer cell line but a weaker inhibitory activity (i.e., ca. 100 μM IC_50_) against the KM12 and MCF7 cancer cell lines, respectively. In contrast, only neopapillarine (**4**) and 4′-senecioyloxyosthol (**5**) showed inhibitory activity with an IC_50_ of 20 μM in the UO31 renal cancer cell line. While neopapillarine (**4**) displayed a moderate inhibitory activity with an IC_50_ of 67 μM in the A498 cell line, 4′-senecioyloxyosthol (**5**) showed relatively weak activity in the A498 cell line, this difference may be due to the nitrogen bearing ester group of neopapillarine (**4**).

**Figure 4:**
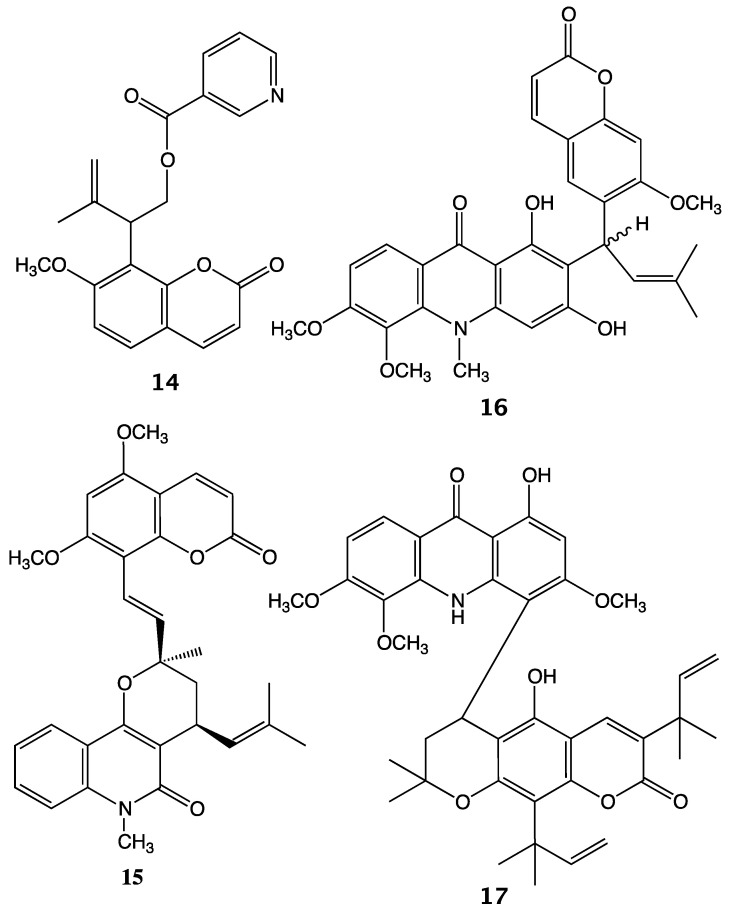
Examples of coumarino-alkaloid compounds isolated from the Rutaceae plants.

**Figure 5:**
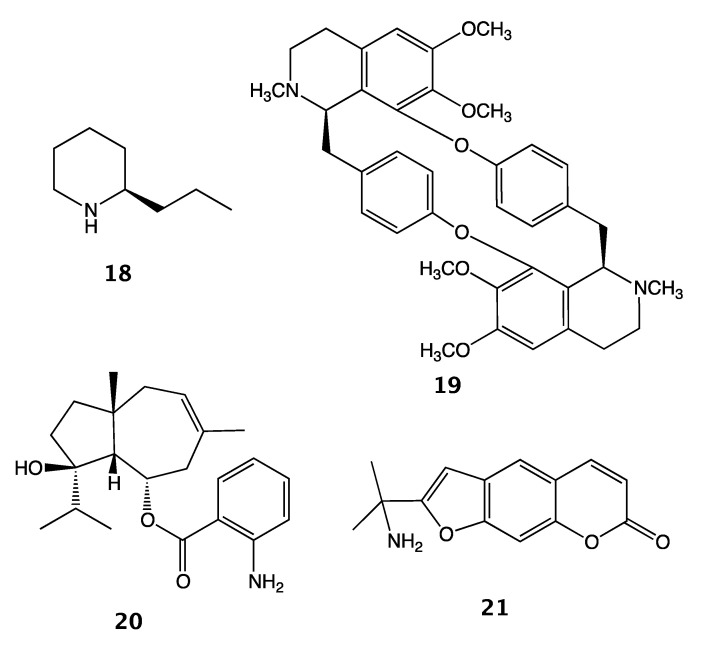
Examples of alkaloids and protoalkaloids isolated from Apiaceae plants.

To date, isomurralonginol nicotinate (**14**) was the only other known nicotinic acid ester of a prenylated coumarin isolated from a plant species of the Rutaceae family, i.e., *Murraya paniculata* (L.) Jack, [28]. Interestingly, other coumarino-alkaloids such as toddacoumalone (**15**) [29], dimeric coumarin coupled quinolone, acrimarines (e.g., acrimarine A (**16**)) [30,31] and neoacrimarines (e.g., neoacrimarine A (**17**)) [32,33], dimeric coumarin coupled acridone alkaloids, were all isolated from the plants of the Rutaceae family (Figure 4). The vast majority of alkaloids discovered in the Apiaceae family have been piperidine derivatives (e.g., coniine (**18**), conyhydrine) [34]. Nevertheless, rarely other types of alkaloids such as bisbenzyisoquinoline (e.g., cycleanine (**19**)) [35] and two protoalkaloid derivatives; elaeochytrine A (**20**) (i.e., jaeschkeanadiol anthranilate) [36] and a furanocoumarin derived prangosine (**21**) [37] have been reported in the Apiaceae family (Figure 5).

## 4. Materials and Methods

### 4.1. General Experimental Procedures

UV spectra were recorded on the Shimadzu UV-Vis Spectrophotometer, UV-1700 (Kyoto, Japan). IR spectra (neat) were recorded on the Perkin-Elmer FT-IR Spectrometer, SPECTRUM 2000 (Waltham, MA, USA). NMR spectra were acquired on a Bruker Avance III spectrometer (Billerica, MA, USA) operating at 600 MHz for ^1^H and 150 MHz for ^13^C and equipped with a 3 mm cryogenically cooled probe. HRESIMS data were acquired on an Agilent 6530 Accurate Mass Q-TOF instrument (Santa Clara, CA, USA). Initial purification of the dichloromethane extract was carried out on a Sephadex LH-20 (GE Healthcare, Chicago, IL, USA) column. Further purification of column fractions was performed using silica gel F254 PLC plates (1 mm thickness) (Merck KGaA, Darmstadt, Germany).

### 4.2. Plant Material

**Figure 6:**
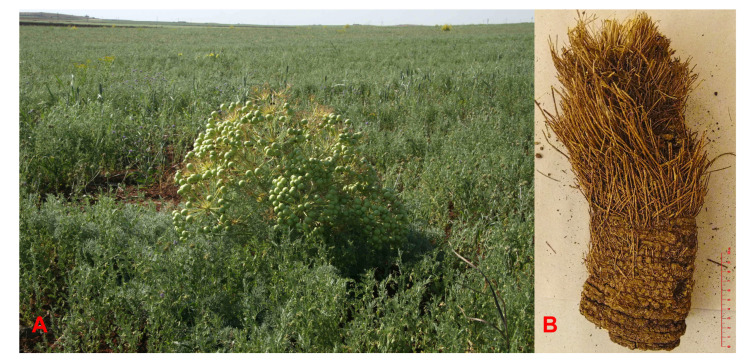
(**A**) A *Neocryptodiscus papillaris* plant in its natural location, (**B**)Piece of the root used in this study (a scale of 10 cm was shown on the lower right side of the root to illustrate its size).

The root and fruits of *N. papillaris* were collected from Aşağı Dilimli village, near Viranşehir, Şanlıurfa in June 2013 and identified by Prof. A. Duran. A voucher specimen (A. Duran 7780) was deposited in the Herbarium of Selçuk University, Faculty of Sciences, Department of Biology. Due to the endangered rare plant species status of *Neocryptodiscus papillaris*, only a portion of the root was taken from the plant (Figure 6), the root material was cut into narrow slices and dried in a well-ventilated area protected from sun light along with the fruits of plant.

### 4.3. Extraction and Isolation

Upon complete drying (*ca.* 1 month) of the plant material, coarsely powdered root (200 g) and fruits (100 g) of *N. papillaris* were extracted sequentially with dichloromethane (4 L for the root and 2 L for the fruits) and methanol (4 L for the root and 2 L for the fruits) at room temperature and concentrated, in vacuo, to dryness (yields; 9.85 g for the dichloromethane root extract, 1.59 g for the dichloromethane fruit extract, 16.72 g for the methanol root extract and 4.22 g for the methanol fruit extract). The dichloromethane root extract (4 g) was separated using a Sephadex LH-20 column (4.5 × 100 cm) packed in a hexane/dichloromethane/methanol (14:9:1) mixture followed by prep. TLC (1 mm thickness, silica gel F254 developed with hexane/ethyl acetate at 9:1, 7:3, or 1:1) for final purification of compounds. The known compounds isolated from the dichloromethane extract were osthol (**1**, 1.08 g), 4′-senecioyloxyosthol (**5**, 24 mg), psoralen (**6**, 6 mg), bergapten (**7**, 3 mg), isoimperatorin (**8**, 32 mg), oxypeucedanin (**9**, 22.5 mg), oxypeucedanin hydrate (**10**, 4.4 mg), pranferol (**11**, 3 mg), scopoletin (**12**, 8 mg) and scoparone (**13**, 1.2 mg).

4′-Hydroxyosthol (**2,** 2.2 mg). White amorphous powder; IR (NaCl) ν_max_: 3450, 2918, 2850, 1730, 1607, 1498, 1403, 1281, 1252, 1120, 1091, 832 cm^−1^; UV (MeOH) λ_max_ (log ε): 321 (3.80), 257 (3.39), 248 (sh) (3.40), 214 (3.94) nm; ^1^H and ^13^C-NMR (see Table 1); HRESIMS *m*/*z* 261.1115 [M + H]^+^ (calcd. for C_15_H_16_O_4_ 261.1121, err. 2.55 ppm).

4′-Acetoxyosthol (**3**, 1.6 mg). White amorphous powder; IR (NaCl) ν_max_: 2933, 2850, 1742, 1730, 1609, 1498, 1436, 1403, 1367, 1281, 1251, 1118, 1090, 1028, 833 cm^−1^; UV (MeOH) λ_max_ (log ε): 322 (3.91), 257 (3.51), 248 (3.50), 210 (3.99) nm; ^1^h and ^13^C-NMR (see Table 1); HRESIMS *m*/*z* 325.1039 [M + Na ^+^ (calcd. for C_17_H_18_O_5_Na 325.1052, err. 4.03 ppm).

Neopapillarine (**4**, 8.1 mg). White amorphous powder; IR (NaCl) ν_max_: 2919, 2850, 1723, 1608, 1498, 1436, 1281, 1251, 1161, 1117, 1089, 1024, 833, 742, 703 cm^−1^; UV (MeOH) λ_max_ (log ε): 321 (3.23), 271 (sh) (2.98), 258 (3.11), 190 (sh) (3.63) nm; ^1^h and ^13^C-NMR (see Table 1); HRESIMS *m*/*z* 366.1337 [M + H]^+^ (calcd. for C_21_H_20_NO_5_ 366.1341, err. 1.23 ppm).

### 4.4. Cytotoxicity Assay on Renal Cancer Cell Lines

The assay used for this study was a two-day, two cell line XTT bioassay [38], an in vitro antitumor colorimetric assay developed by the MTP Assay Development and Screening Section. The renal cancer cell lines used were UO31 and A498. Colon cancer cell lines were COLO205 and KM12, and MCF-7 was the breast cancer cell line. Cells were harvested and plated (45 μL) at a seeding density of 3.0 × 10^5^ cells per well for the UO31 cell line, 2.5 × 10^5^ cells per well for the A498 cell line, 3.5 × 10^5^ cells per well for the COLO205 and KM12 colon cancer cell lines, and 3.0 × 10^5^ cells per well for the MCF7 breast cancer cell line. The respective cell lines were separately plated into 384-well assay plates and then incubated for 24 h. DMSO solutions of the test materials (8 μL) were diluted 1:25 with medium (192 μL) and then subjected to five 2:1 serial dilutions (100 μL each) on a 96-well plate. Duplicate 40 μL aliquots of each sample concentration were then transferred to a 384-well “dilution plate”, which could accommodate the duplicate samples from two 96-well plates. A 5 μL portion of each solution in the dilution plate was transferred to the cell cultures in the 384-well assay plates to give a final volume of 50 μL and a DMSO concentration of 0.4%. Control wells included 8 wells with the positive control sanguinarine chloride at 20 µM, as well as DMSO only controls and no cell controls. The Z’ factors for the individual plates were calculated and were >0.5 in all cases. The cells were incubated for 48 h at 37 °C in the presence of the test samples and then treated with the tetrazolium salt XTT (2,3-bis[2-methoxy-4-nitro-5-sulfophenyl]-2h-tetrazolium-5-carboxanilide). Viable cells reduced XTT to a colored formazan product, and after an additional 4 h incubation period the amount of formazan produced was quantified by absorption at 450 nm, using a 650 nm reference. Plates were read on a PerkinElmer EnVision (model # 2104) reader.

## Figures and Tables

**Table 1:** ^1^H-NMR and ^13^C-NMR data for Compounds **2, 3,** and **4** in CDCl_3_.

	Compound 2		Compound 3		Compound 4	
Pos.	δ_H_ (in ppm, m, *J* in Hz)	δ_C_ (in ppm, type)	δ_H_ (in ppm, m, *J* in Hz)	δ_C_ (in ppm, type)	δ_H_ (in ppm, m, *J* in Hz)	δ_C_ (in ppm, type)
2		162.09; C		161.24; C		161.21; C
3	6.25; d; 9.5; 1H	112.96; CH	6.23; d; 9.4; 1H	113.24, CH	6.23; d; 9.5; 1H	127.70; CH
4	7.65; d; 9.5; 1H	144.32; CH	7.61; d; 9.4; 1H	143.81; CH	7.61; d; 9.5; 1H	143.82; CH
5	7.33; d; 8.5; 1H	126.76; CH	7.31; d; 8.6; 1H	126.73; CH	7.31; d; (8.6); 1H	126.82; CH
6	6.86; d; 8.5; 1H	107.80; CH	6.82; d; 8.6; 1H	107.46; CH	6.83; d; 8.6; 1H	107.49; CH
7		160.01; C		160.19; C		160.19; C
8		116.70; C		116.68; C		116.56; C
9		153.39; C		153.03; C		153.01; C
10		113.50; C		113.11; C		113.15; C
OCH_3_	3.94; s; 3H	56.36; CH_3_	3.91; s; 3H	56.18; CH_3_	3.90; s; 3H	56.24; CH_3_
1′	3.59; br dd; 0.6, 7.9; 2H	21.63; CH_2_	3.61; br d; 7.7; 2H	21.73; CH_2_	3.68; br d; 7.7; 2H	21.65; CH_2_
2′	5.22; br t; 7.9; 1H	123.14; CH	5.51; br t; 7.7; 1H	126.98; CH	5.60; br t; 7.7; 1H	113.30; CH
3′		136.51; C		130.98; C		130.54; C
4′	4.41; d; 0.6; 2H	61.21; CH_2_	4.85; br s; 2H	63.46; CH_2_	5.16; br s; 2H	64.53; CH_2_
5′	1.80; br d; 1.4; 3H	21.59; CH_3_	1.72; br d; 0.9; 3H	21.52; CH_3_	1.81; br d; 1.0; 3H	21.85; CH_3_
1′′				171.37; C		165.33; C
2′′			2.09; s; 3H	21.14; CH_3_		126.82; C
3′′					8.35; d t; 1.9, 7.8; 1H	137.67; CH
4′′					7.42; br dd; 4.8, 7.8; 1H	123.63; CH
5′′					8.78; br d; 3.5; 1H	153.07; CH
6′′					9.24; br s; 1H	150.71; CH

**Table 2:** Inhibitory concentrations, (IC_50_, µM) values of prenylated coumarins isolated from *N. papillaris*.

Compounds	1	2	3	4	5	8	9
A498	>1000	>1000	>1000	67	>100	>1000	720
UO31	>1000	≥1000	>1000	20	20	>1000	630
COLO205	20	NT *	NT	NT	NT	14	30
KM12	>100	NT	NT	NT	NT	>100	31
MCF7	24	NT	NT	NT	NT	>100	>100

* NT; not tested.

## Supplementary Materials

The following are available online, Figure S1: Structures of the coumarin derivatives isolated from the roots of *Neocryptodiscus papillaris,* Figure S2: ^1^H NMR spectrum (600 MHz, CDCl_3_) of 4′-Hydroxyosthol (**2**), Figure S3: ^13^C NMR spectrum (125 MHz, CDCl_3_) of 4′-Hydroxyosthol (**2**), Figure S4: 2D-COSY spectrum of 4′-Hydroxyosthol (**2**), Figure S5: 2D HSQC spectrum 4′-Hydroxyosthol (**2**), Figure S6: 2D HMBC spectrum of 4′-Hydroxyosthol (**2**), Figure S7: 2D NOESY spectrum of 4′-Hydroxyosthol (**2**), Figure S8: HRESIMS spectrum of 4′-Hydroxyosthol (**2**), Figure S9: ^1^H NMR spectrum (600 MHz, CDCl_3_) of 4′-Acetoxyosthol (**3**), Figure S10: ^13^C NMR spectrum (125 MHz, CDCl_3_) of 4′-Acetoxyosthol (**3**), Figure S11: 2D-COSY spectrum of 4′-Acetoxyosthol (**3**), Figure S12: 2D HSQC spectrum 4′-Acetoxyosthol (**3**), Figure S13: 2D HMBC spectrum of 4′-Acetoxyosthol (**3**), Figure S14: 2D NOESY spectrum of 4′-Acetoxyosthol (**3**), Figure S15: HRESIMS spectrum of 4′-Acetoxyosthol (**3**), Figure S16: ^1^H NMR spectrum (600 MHz, CDCl_3_) of Neopapillarine (**4**), Figure S17: ^13^C NMR spectrum (125 MHz, CDCl3) of Neopapillarine (**4**). Figure S18: 2D-COSY spectrum of Neopapillarine (**4**), Figure S19: 2D HSQC spectrum Neopapillarine (**4**), Figure S20: 2D HMBC spectrum of Neopapillarine (**4**), Figure S21: 2D NOESY spectrum of Neopapillarine (**4**), Figure S22: HRESIMS spectrum of Neopapillarine (**4**), Figure S23: ^1^H NMR spectrum of Osthol (**1**), Figure S24: ^1^H NMR spectrum of 4′-Senecioyloxyosthol (**5**), Figure S25: ^1^H NMR spectrum of Psoralen (**6**), Figure S26: ^1^H NMR spectrum of Bergapten (**7**), Figure S27: ^1^H NMR spectrum of Isoimperatorin (**8**), Figure S28: ^1^H NMR spectrum of Oxypeucedanin (**9**), Figure S29: ^1^H NMR spectrum of Oxypeucedanin Hydrate (**10**), Figure S30: ^1^H NMR spectrum of Pranferol (**11**), Figure S31: ^1^H NMR spectrum of Scopoletin (**12**), Figure S32: ^1^H NMR spectrum of Scoparone (**13**).
