# Supplementary material for: Neopapillarine, an Unusual Coumarino-Alkaloid from the Root Extract of Neocryptodiscus papillaris with Cytotoxic Activity on Renal Cancer Cells
Source: Molecules. 2020 Jul 3;25(13):3040. doi: 10.3390/molecules25133040 (PMC7420726; doi:10.3390/molecules25133040)
Supplement: Supplementary file 1 [file molecules-25-03040-s001.pdf]

Supplementary Materials

Neopapillarine, an Unusual Coumarino-alkaloid from the Root Extract of *Neocryptodiscus papillaris* with Selective Cytotoxic Activity on Renal Cancer Cells

Fatma Tosun[1], Feyyaz Mıhoğlugil[2], John A. Beutler[3], Esra Eroğlu Özkan[3,4] and Mahmut Miski[4]

Affiliations

[1] Istanbul Medipol University, School of Pharmacy, Department of Pharmacognosy, Istanbul 34810, Turkey

[2] Cyprus International University, Faculty of Pharmacy, Nicosia 99258, North Cyprus

[3] Molecular Targets Program, Center for Cancer Research, National Cancer Institute, Frederick, MD 21702, USA

[4] Istanbul University, Faculty of Pharmacy, Department of Pharmacognosy, Istanbul 34116, Turkey

Correspondence

Prof. Dr. Mahmut Miski, Istanbul University, Department of Pharmacognosy, Istanbul 34116, Turkey

mahmud.miski@istanbul.edu.tr; Phone: +90-545-550-4455 Fax: +90-212-440-0252

Table of Contents

Fig. S1 Structures of the coumarin derivatives isolated from the roots of *Neocryptodiscus papillaris*....3
Fig. S2 ^{1}H NMR spectrum (600 MHz, CDCl3) of 4′-Hydroxyosthol (**2**)....4
Fig. S3 ^{13}C NMR spectrum (125 MHz, CDCl3) of 4′-Hydroxyosthol (**2**)....5
Fig. S4 2D COSY spectrum of 4′-Hydroxyosthol (**2**)....6
Fig. S5 2D HSQC spectrum 4′-Hydroxyosthol (**2**)....7
Fig. S6 2D HMBC spectrum of 4′-Hydroxyosthol (**2**)....8
Fig. S7 2D NOESY spectrum of 4′-Hydroxyosthol (**2**)....9
Fig. S8 HRESIMS spectrum of 4′-Hydroxyosthol (**2**)....10
Fig. S9 ^{1}H NMR spectrum (600 MHz, CDCl3) of 4′-Acetoxyosthol (**3**)....11
Fig. S10 ^{13}C NMR spectrum (125 MHz, CDCl3) of 4′-Acetoxyosthol (**3**)....12
Fig. S11 2D COSY spectrum of 4′-Acetoxyosthol (**3**)....13
Fig. S12 2D HSQC spectrum 4′-Acetoxyosthol (**3**)....14
Fig. S13 2D HMBC spectrum of 4′-Acetoxyosthol (**3**)....15
Fig. S14 2D NOESY spectrum of 4′-Acetoxyosthol (**3**)....16
Fig. S15 HRESIMS spectrum of 4′-Acetoxyosthol (**3**)....17
Fig. S16 ^{1}H NMR spectrum (600 MHz, CDCl3) of Neopapillarine (**4**)....18
Fig. S17 ^{13}C NMR spectrum (125 MHz, CDCl3) of Neopapillarine (**4**)....19
Fig. S18 2D COSY spectrum of Neopapillarine (**4**)....20
Fig. S19 2D HSQC spectrum Neopapillarine (**4**)....21
Fig. S20 2D HMBC spectrum of Neopapillarine (**4**)....22
Fig. S21 2D NOESY spectrum of Neopapillarine (**4**)....23
Fig. S22 HRESIMS spectrum of Neopapillarine (**4**)....24
Fig. S23 ^{1}H NMR spectrum of Osthol (**1**)....25
Fig. S24 ^{1}H NMR spectrum of 4′-Senecioyloxyosthol (**5**)....26
Fig. S25 ^{1}H NMR spectrum of Psoralen (**6**)....27
Fig. S26 ^{1}H NMR spectrum of Bergapten (**7**)....28
Fig. S27 ^{1}H NMR spectrum of Isoimperatorin (**8**)....29
Fig. S28 ^{1}H NMR spectrum of Oxypeucedanin (**9**)....30
Fig. S29 ^{1}H NMR spectrum of Oxypeucedanin Hydrate (**10**)....31
Fig. S30 ^{1}H NMR spectrum of Pranferol (**11**)....32
Fig. S31 ^{1}H NMR spectrum of Scopoletin (**12**)....33
Fig. S32 ^{1}H NMR spectrum of Scoparone (**13**)....34

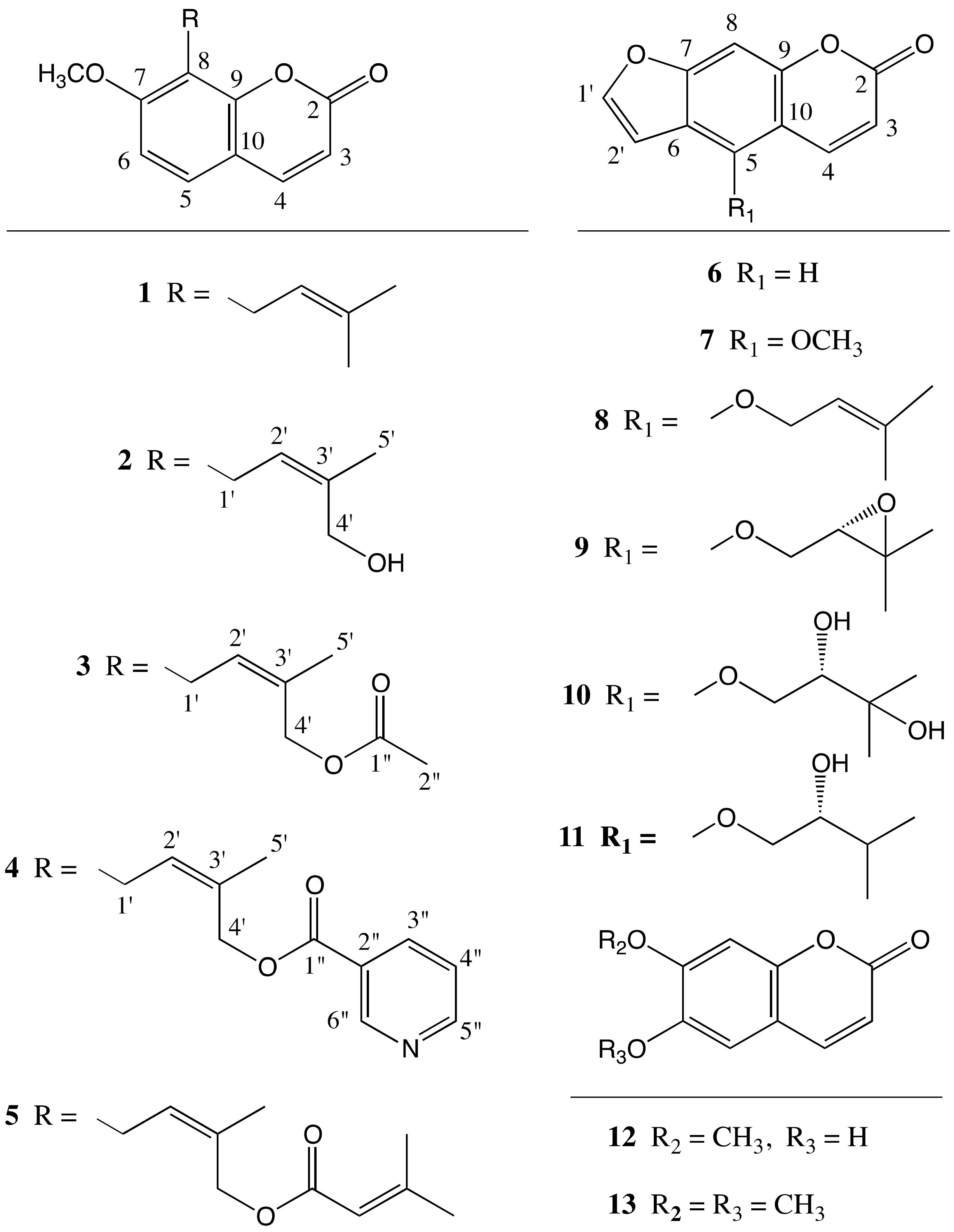

Fig. S1 Structures of the coumarin derivatives isolated from the roots of *Neocryptodiscus papillaris*

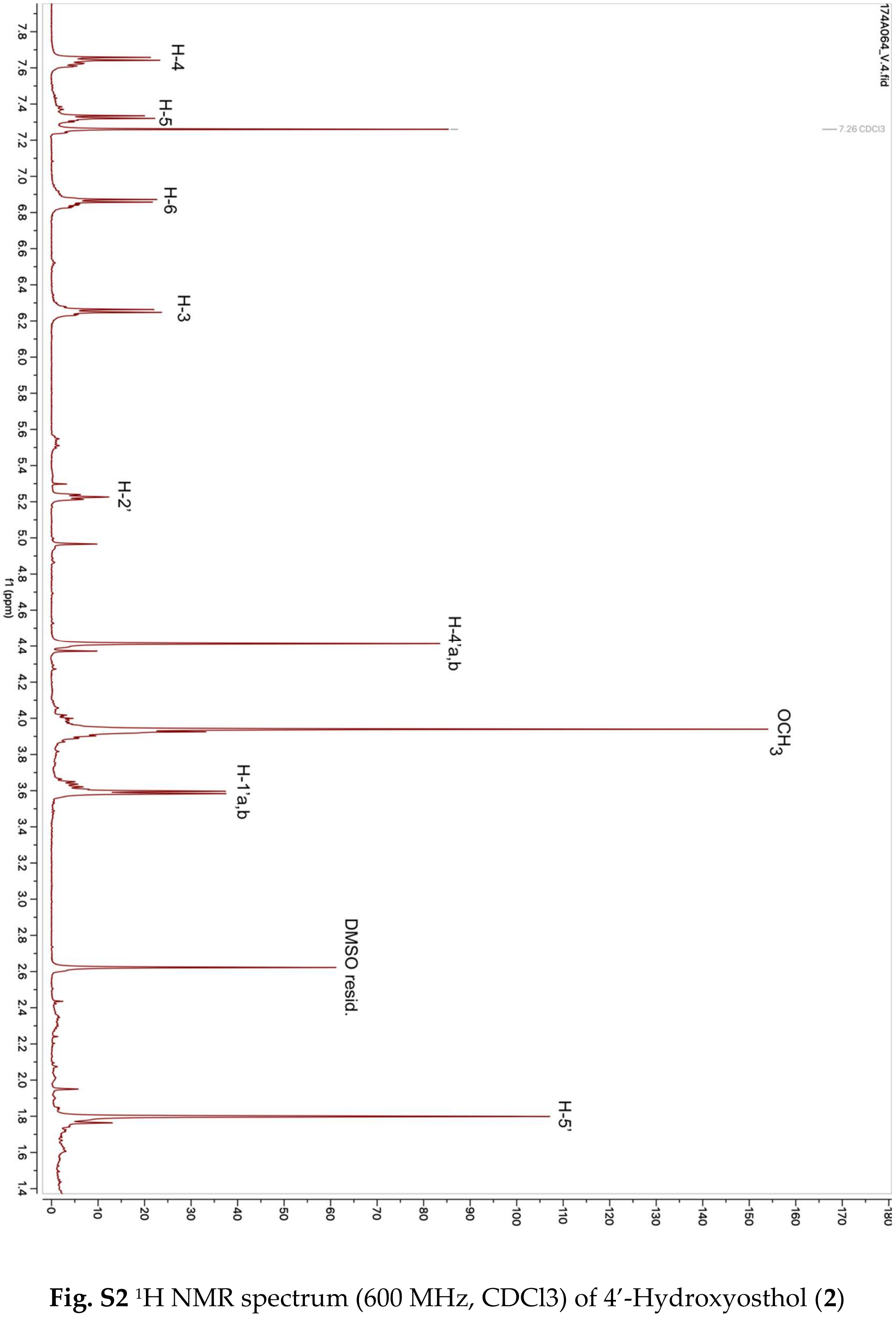

Fig. S2 ^{1}H NMR spectrum (600 MHz, CDCl3) of 4′-Hydroxyosthol (**2**)

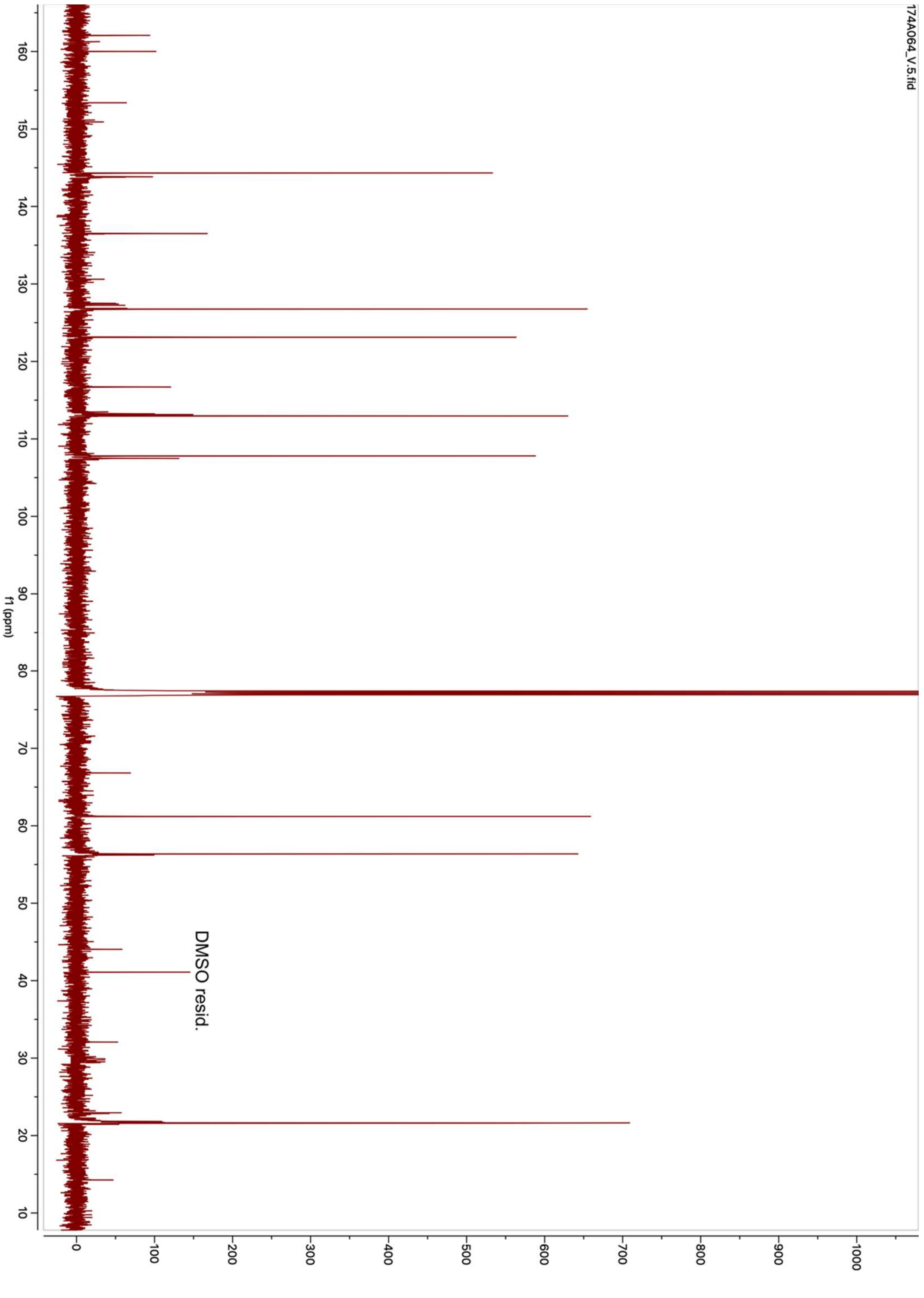

Fig. S3 ^{13}C NMR spectrum (125 MHz, CDCl3) of 4′-Hydroxyosthol (**2**)

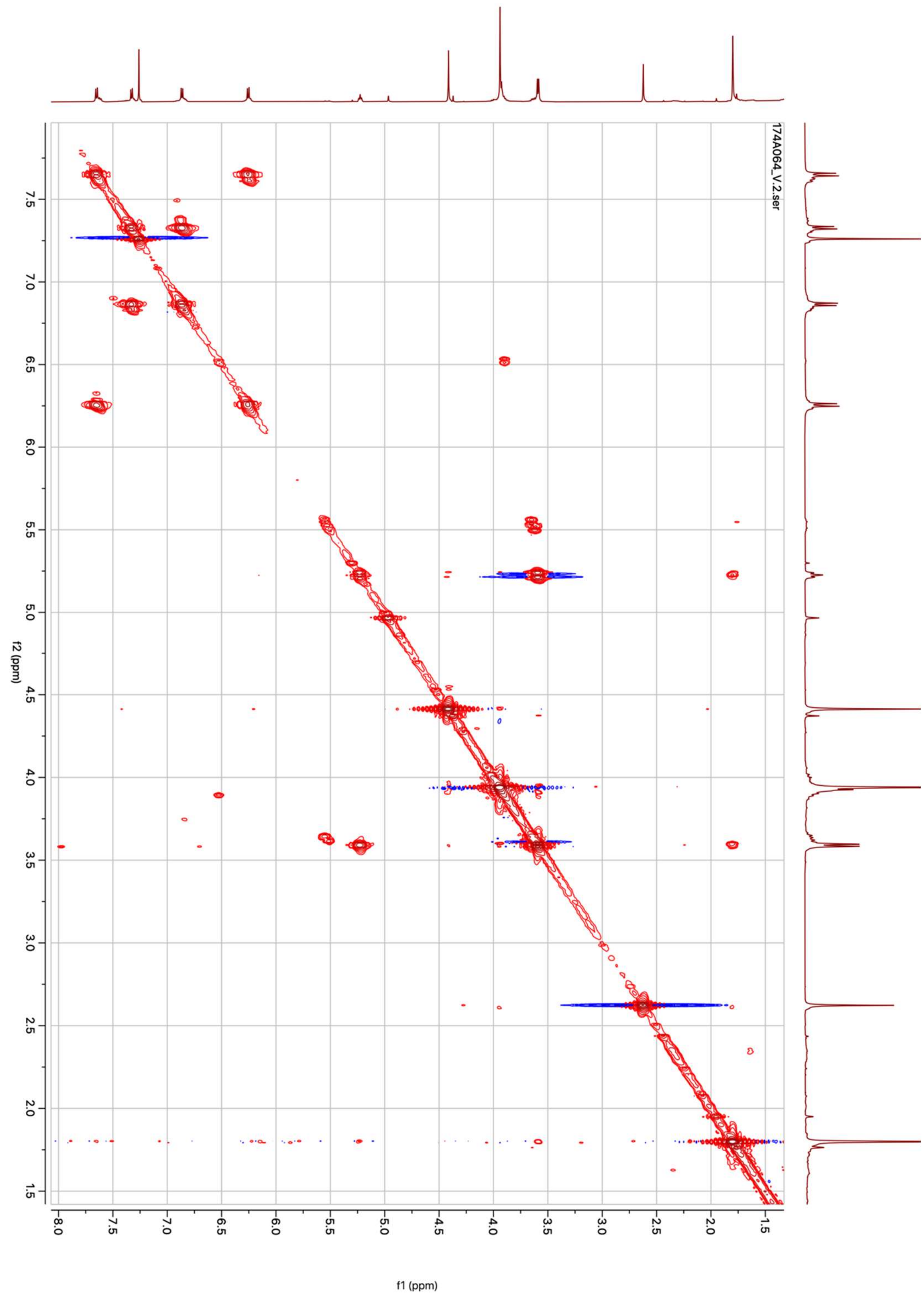

Fig. S4 2D COSY spectrum of 4′-Hydroxyosthol (**2**)

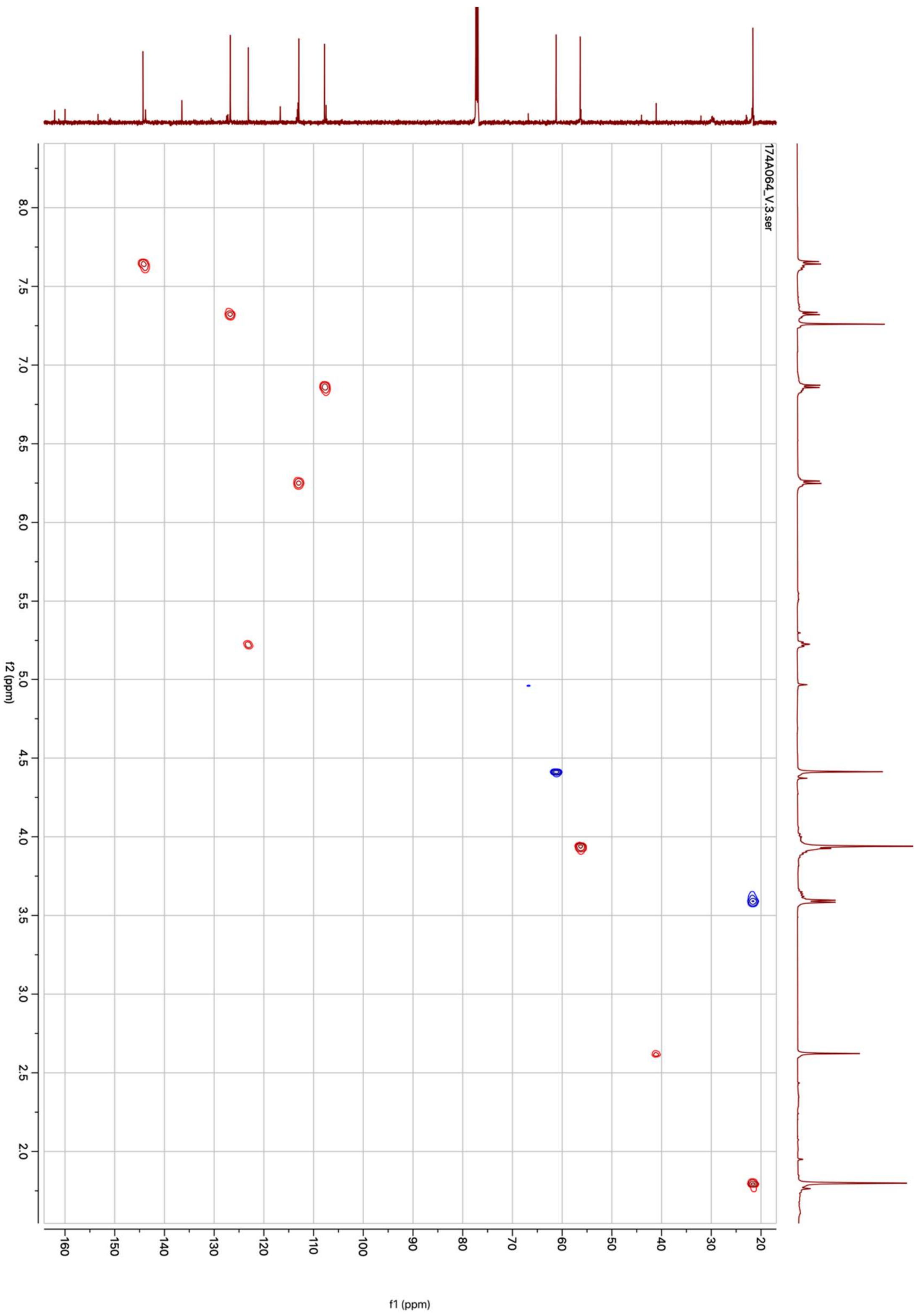

Fig. S5 2D HSQC spectrum 4′-Hydroxyosthol (**2**)

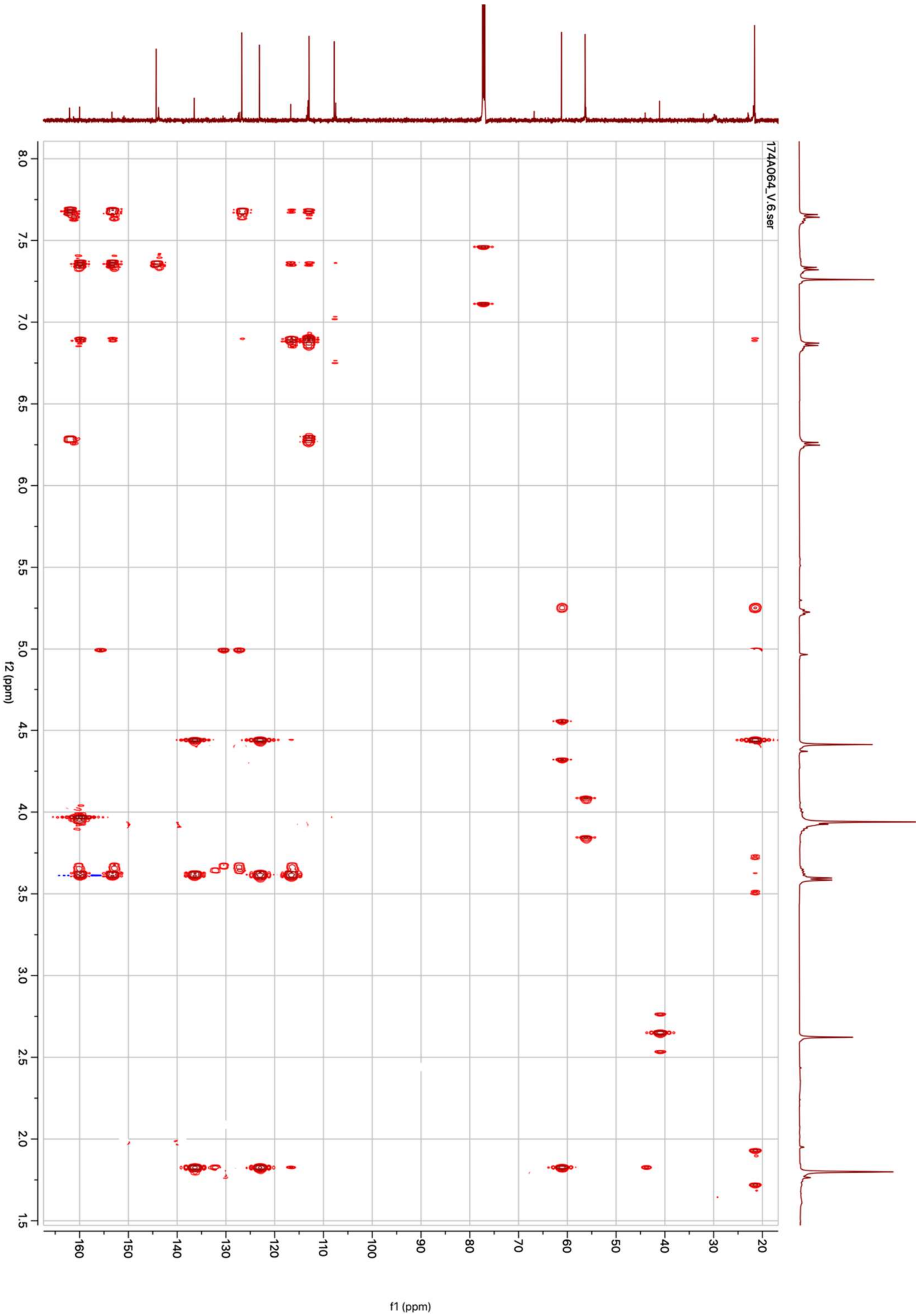

Fig. S6 2D HMBC spectrum of 4′-Hydroxyosthol (**2**)

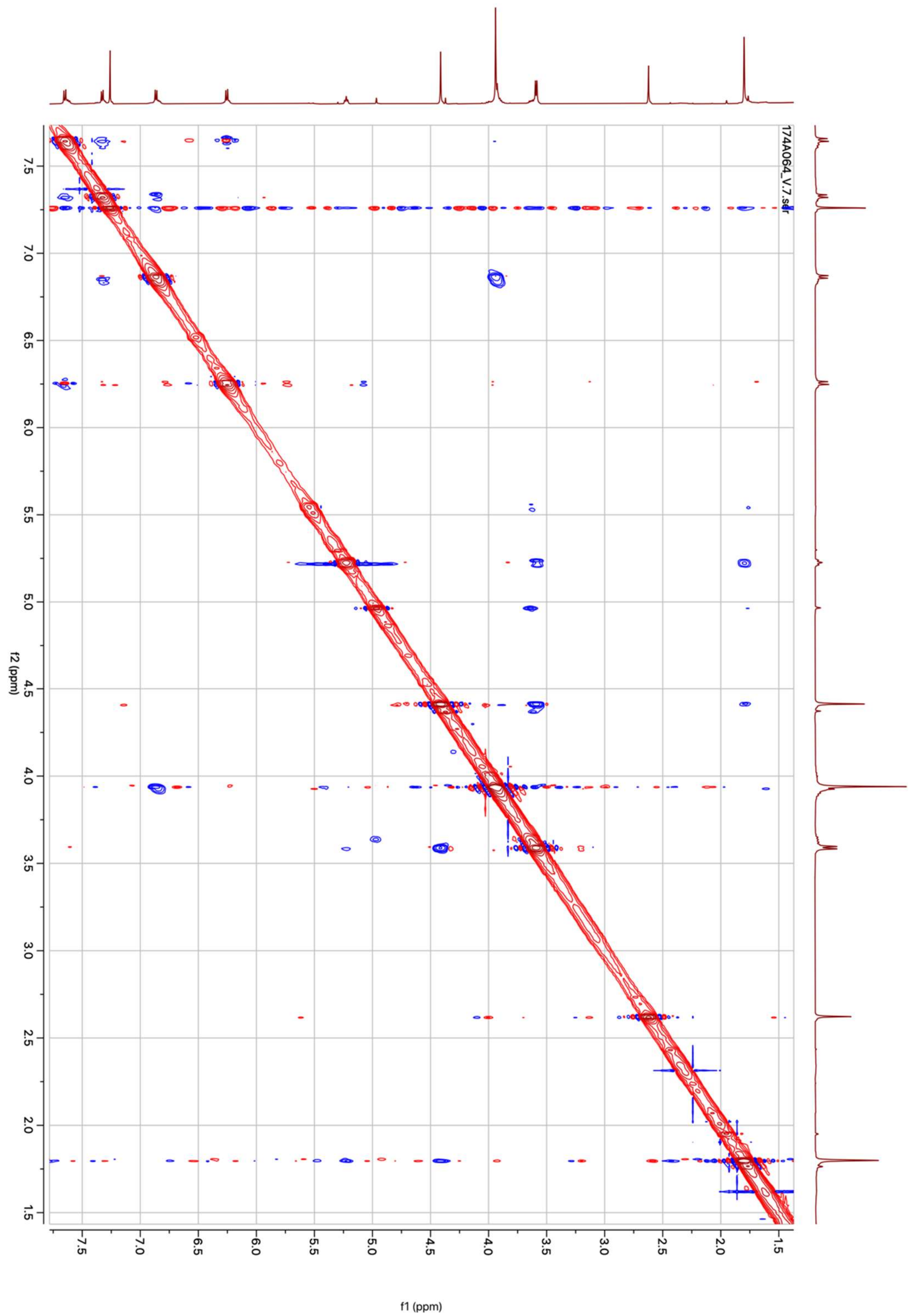

Fig. S7 2D NOESY spectrum of 4′-Hydroxyosthol (**2**)

Qualitative Compound Report

**Data File**	174A064V_dil.d	**Sample Name**	174A064V_dil
**Sample Type**	Sample	**Position**	Vial 22
**Instrument Name**	Instrument 1	**User Name**	Heidi
**Acq Method**	SMaccms_DESI(+)_Centroid.m	**Acquired Time**	2/13/2018 2:13:04 PM
**IRM Calibration Status**	Success	**DA Method**	Default.m
**Comment**			

**Sample Group**		**Info.**	
**Stream Name**	LC 1	**Acquisition SW Version**	6200 series TOF/6500 series Q-TOF B.06.01 (B6172 SP1)

Compound Table

Compound Label	RT	Mass	Formula	MFG Formula	MFG Diff (ppm)	DB Formula
Cpd 5: C15 H16 O4; 283.09237 260.10420	4.93	260.1042	C15 H16 O4	C15 H16 O4	2.55	C15 H16 O4

Compound Label	*m/z*	RT	Algorithm	Mass
Cpd 5: C15 H16 O4; 283.09237 260.10420	283.09237	4.93	Find by Molecular Feature	260.1042

MFE MS Spectrum

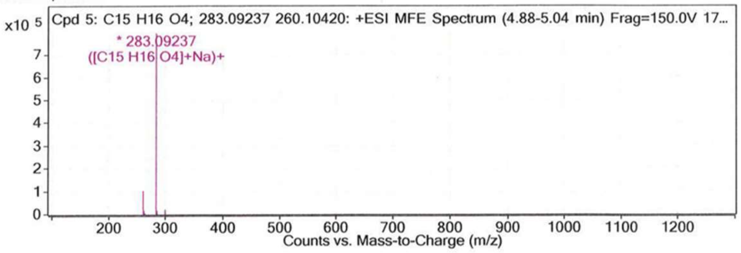

MFE MS Zoomed Spectrum

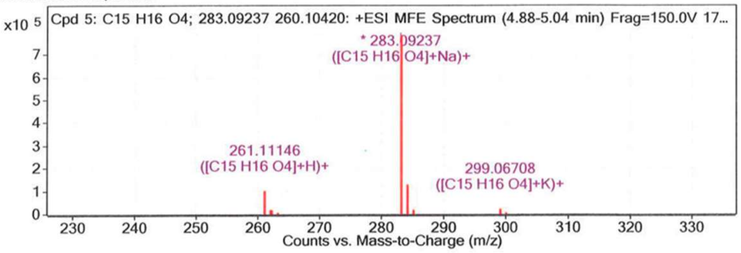

MS Spectrum Peak List

m/z	z	Abund	Formula	Ion
261.11146	1	101057.21	C15 H16 O4	(M+H)+
262.11485	1	15771.74	C15 H16 O4	(M+H)+
263.11772	1	2098.45	C15 H16 O4	(M+H)+
283.09237	1	795776.88	C15 H16 O4	(M+Na)+
284.09707	1	113655.14	C15 H16 O4	(M+Na)+
285.09942	1	15181.58	C15 H16 O4	(M+Na)+

Fig. S8 HRESIMS spectrum of 4′-Hydroxyosthol (**2**)

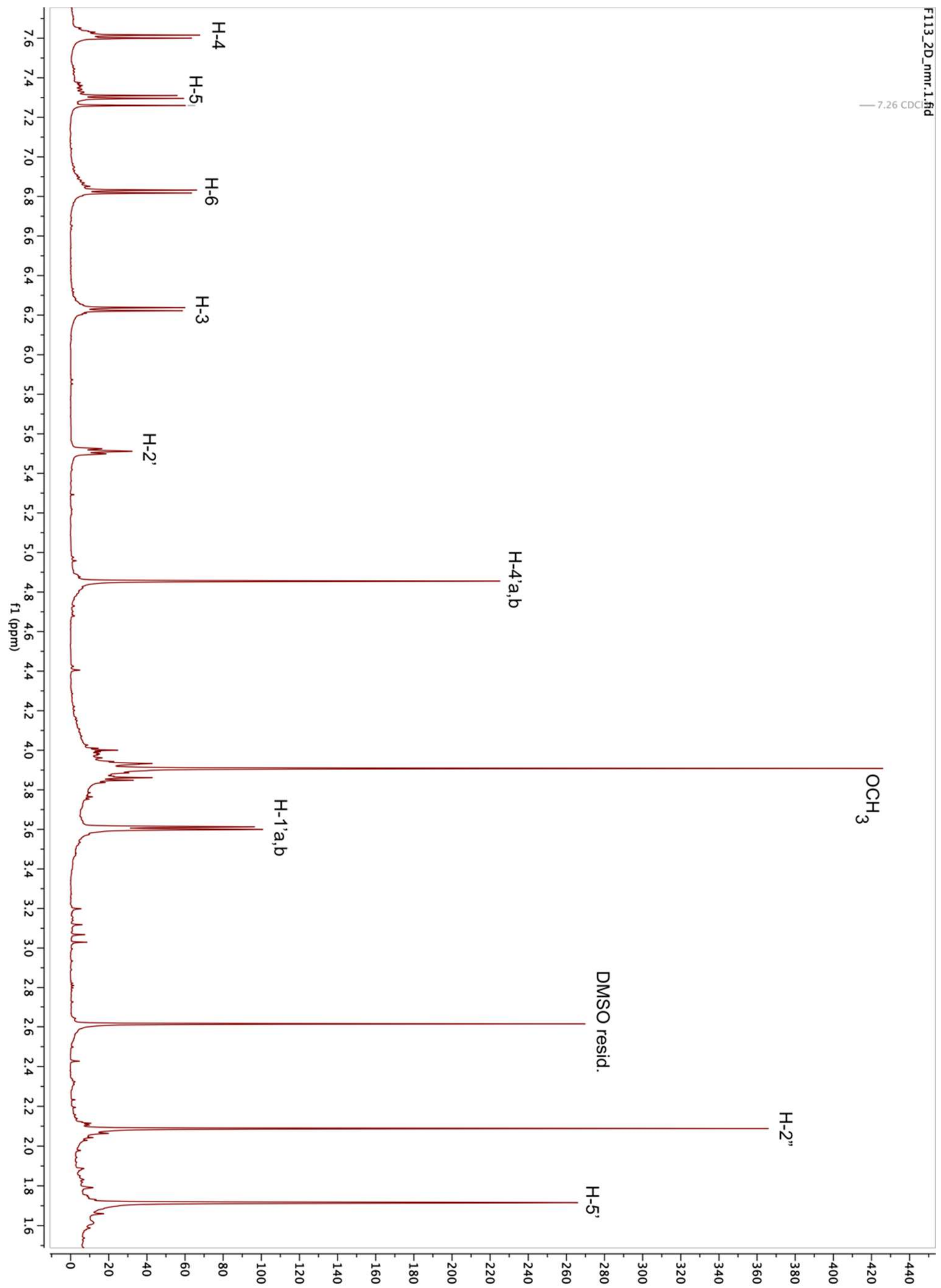

Fig. S9 ^{1}H NMR spectrum (600 MHz, CDCl3) of 4′-Acetoxyosthol (**3**)

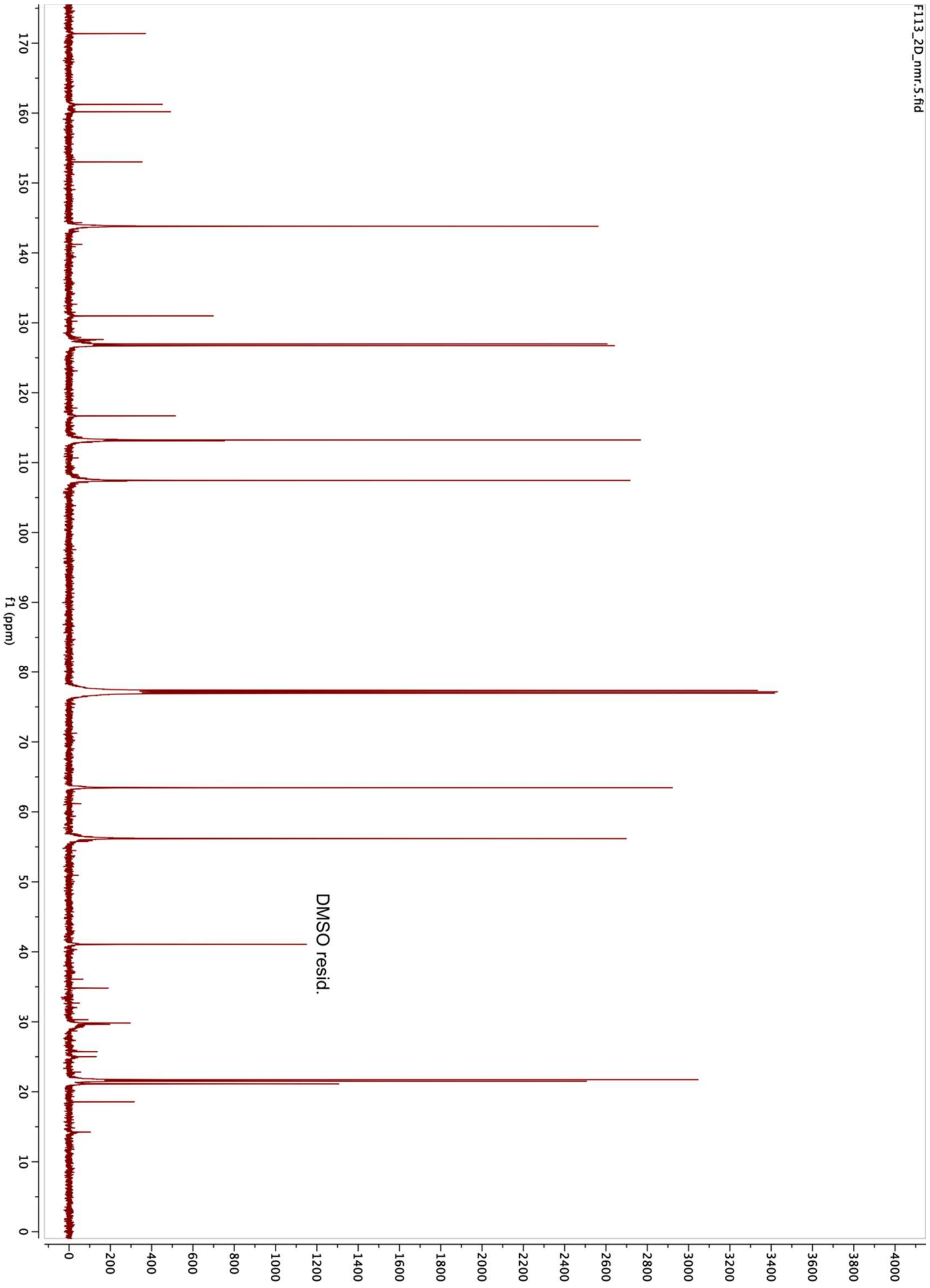

Fig. S10 ^{13}C NMR spectrum (125 MHz, CDCl3) of 4′-Acetoxyosthol (**3**)

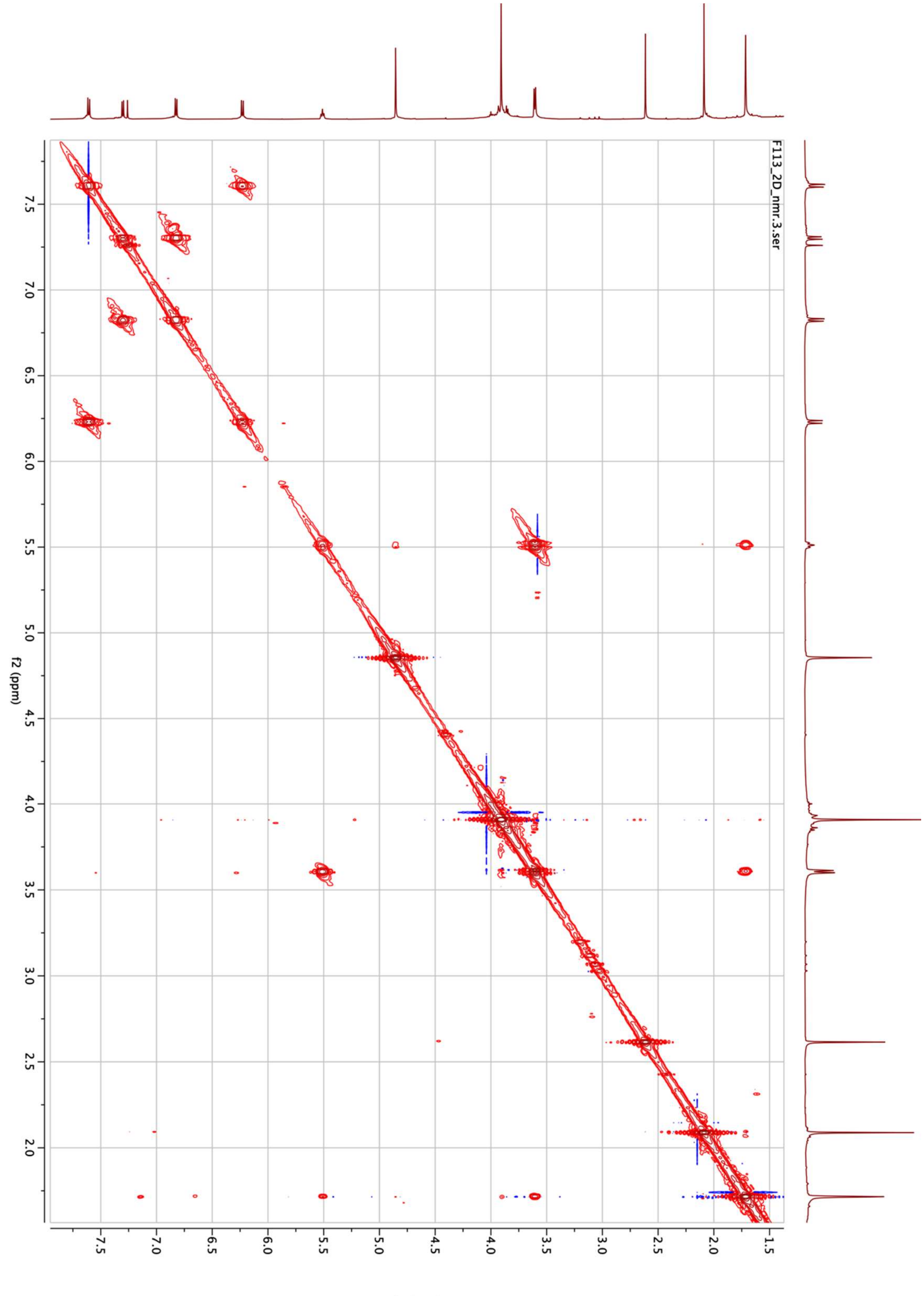

Fig. S11 2D COSY spectrum of 4′-Acetoxyosthol (**3**)

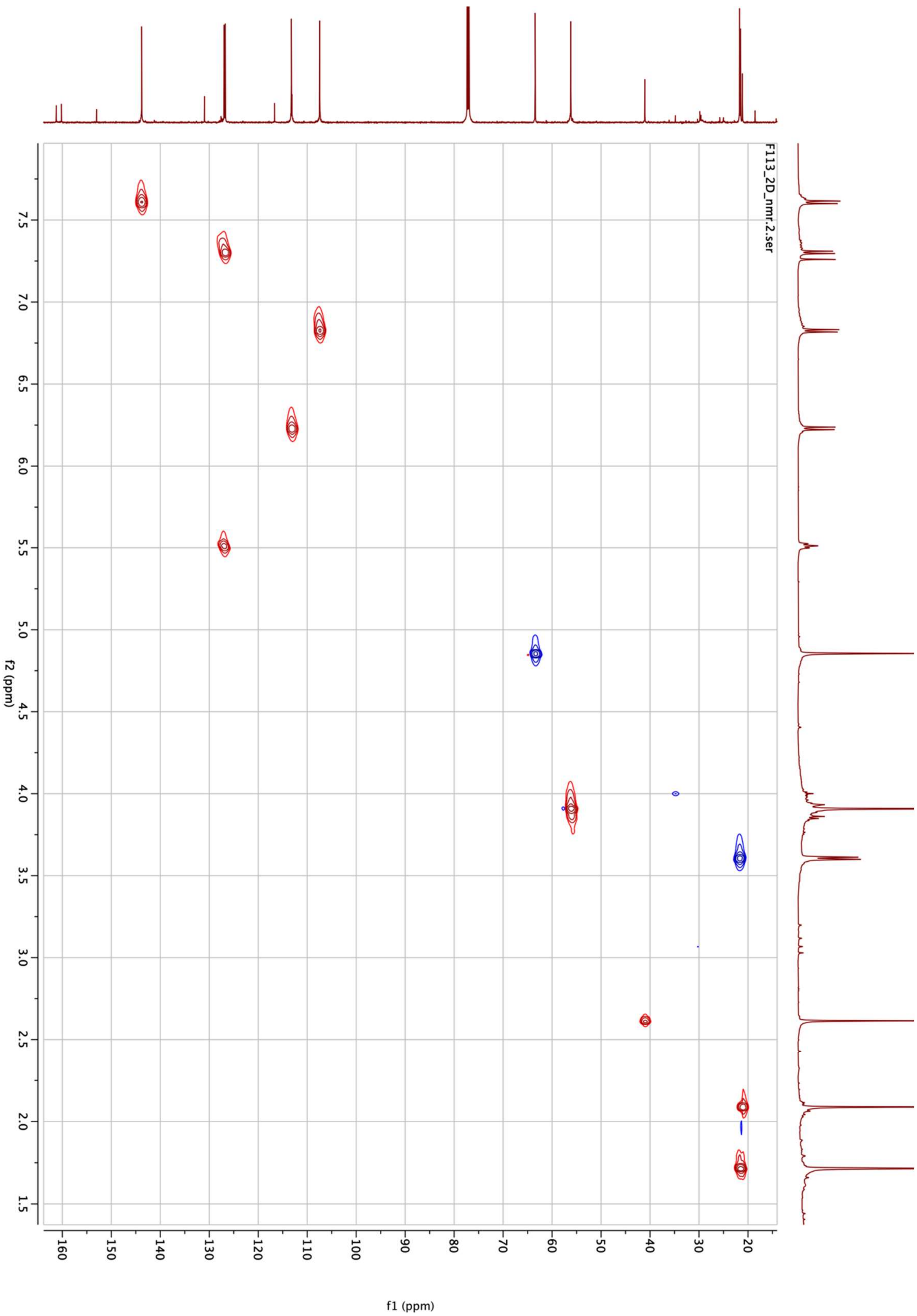

Fig. S12 2D HSQC spectrum 4′-Acetoxyosthol (**3**)

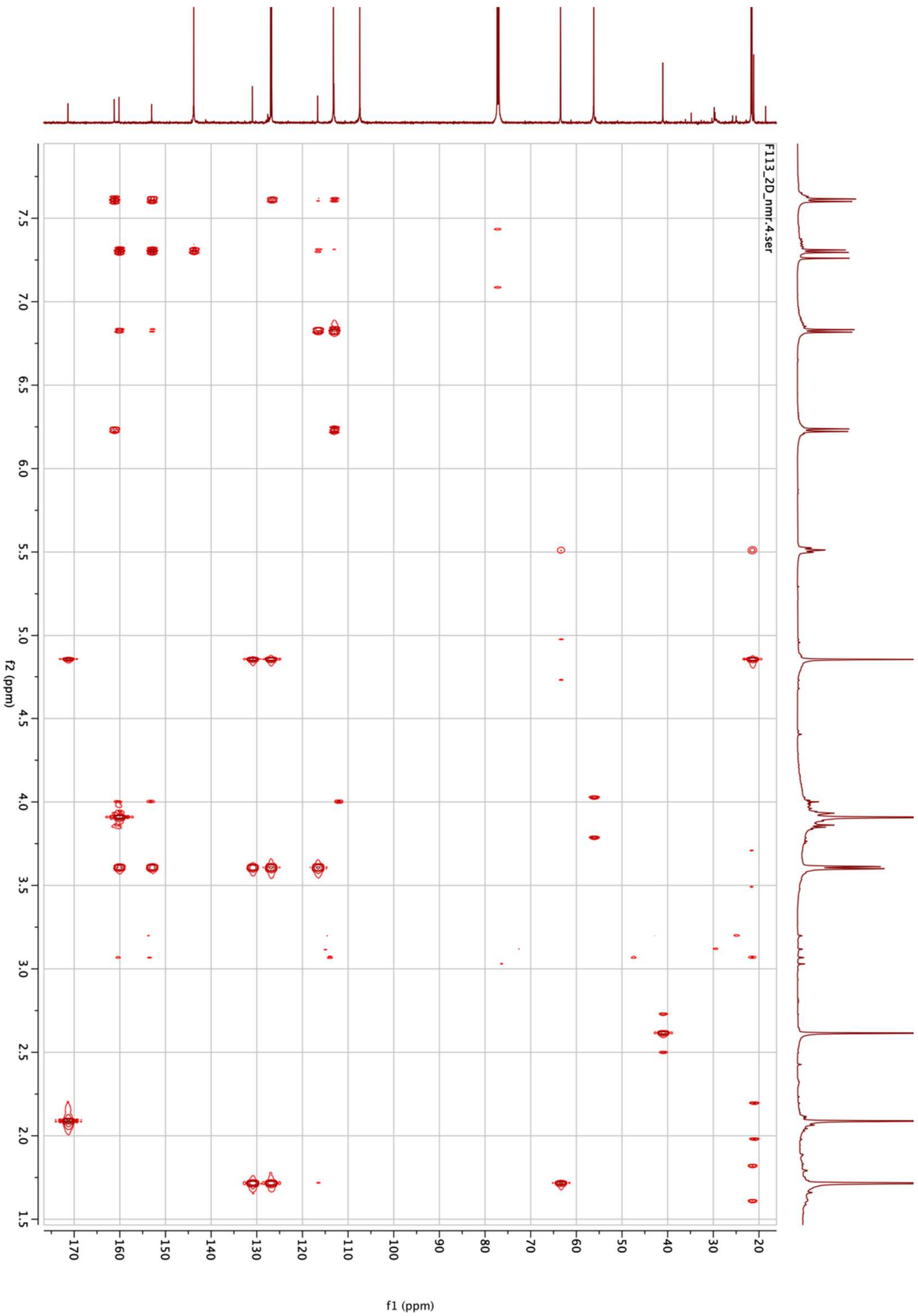

Fig. S13 2D HMBC spectrum of 4′-Acetoxyosthol (**3**)

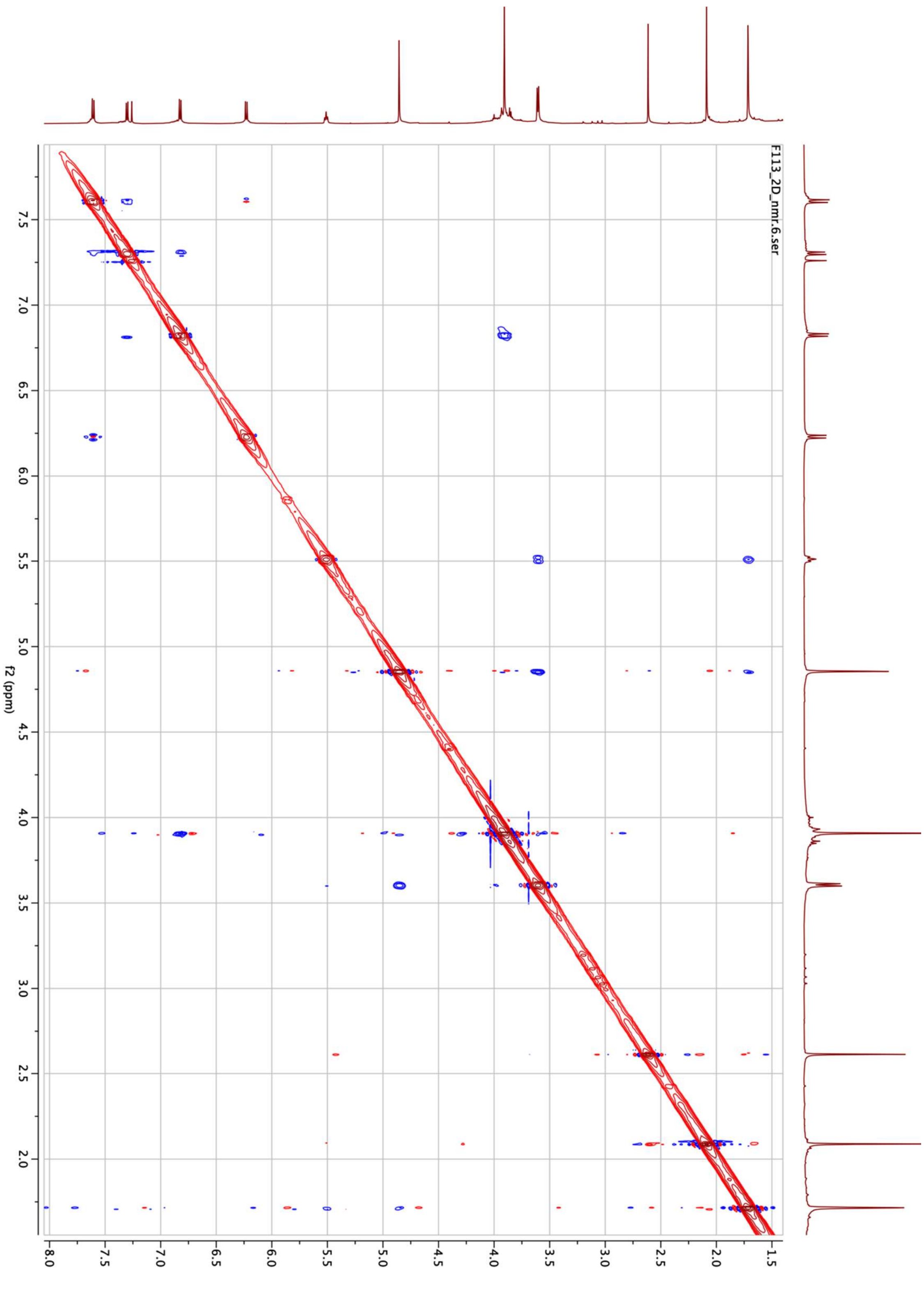

Fig. S14 2D NOESY spectrum of 4′-Acetoxyosthol (**3**)

Qualitative Compound Report

**Data File**	174A064Y_1000.d	**Sample Name**	174A064Y_1000
**Sample Type**	Sample	**Position**	Vial 21
**Instrument Name**	Instrument 1	**User Name**	Heidi
**Acq Method**	SMaccms_DESI(+)_Centroid.m	**Acquired Time**	2/13/2018 4:36:20 PM
**IRM Calibration Status**	Success	**DA Method**	Default.m
**Comment**			

**Sample Group**		**Info.**	
**Stream Name**	LC 1	**Acquisition SW Version**	6200 series TOF/6500 series Q-TOF B.06.01 (B6172 SP1)

Compound Table

Compound Label	RT	Mass	Formula	MFG Formula	MFG Diff (ppm)	DB Formula
Cpd 2: C17 H18 O5; 325.10388 302.11467	5.63	302.11467	C17 H18 O5	C17 H18 O5	2.48	C17 H18 O5

Compound Label	*m/z*	RT	Algorithm	Mass
Cpd 2: C17 H18 O5; 325.10388 302.11467	325.10388	5.63	Find by Molecular Feature	302.11467

MFE MS Spectrum

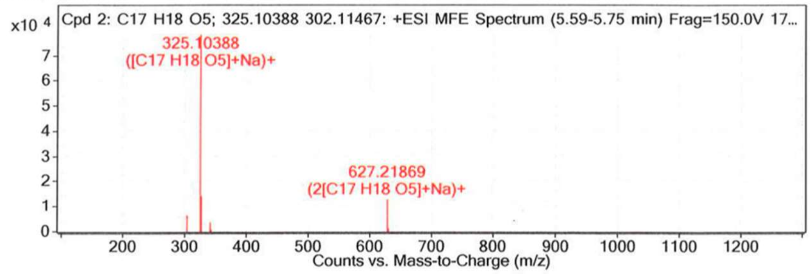

MFE MS Zoomed Spectrum

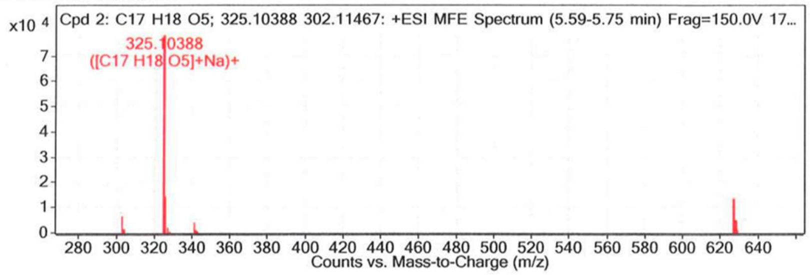

MS Spectrum Peak List

m/z	z	Abund	Formula	Ion
303.12244	1	6281.4	C17 H18 O5	(M+H)+
304.12499	1	1372.62	C17 H18 O5	(M+H)+
325.10388	1	78310.41	C17 H18 O5	(M+Na)+
326.10739	1	14248.89	C17 H18 O5	(M+Na)+
327.11008	1	2089.23	C17 H18 O5	(M+Na)+
341.07719	1	3814.93	C17 H18 O5	(M+K)+

Fig. S15 HRESIMS spectrum of 4′-Acetoxyosthol (**3**)

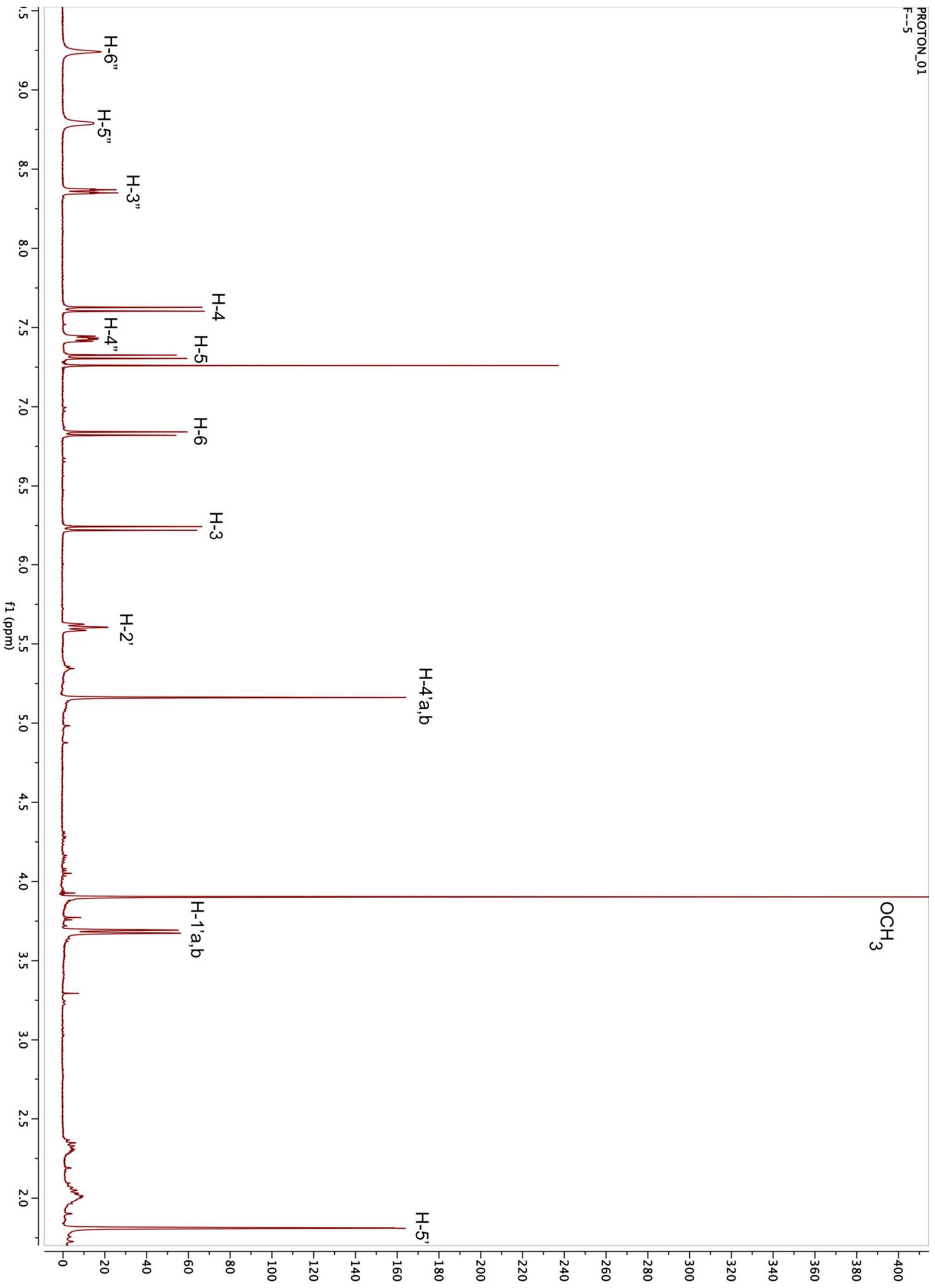

Fig. S16 ^{1}H NMR spectrum (600 MHz, CDCl3) of Neopapillarine (**4**)

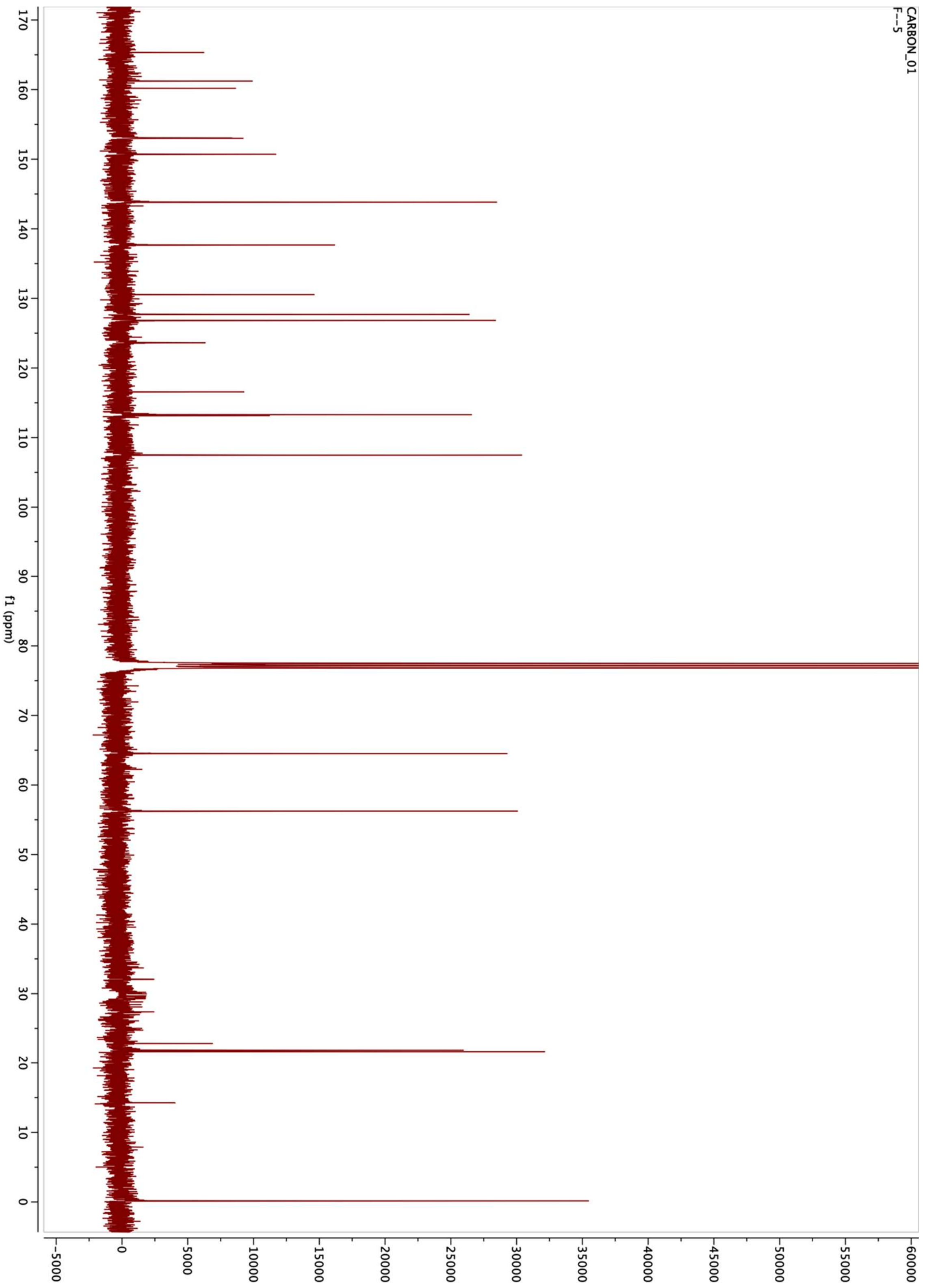

Fig. S17 ^{13}C NMR spectrum (125 MHz, CDCl3) of Neopapillarine (**4**)

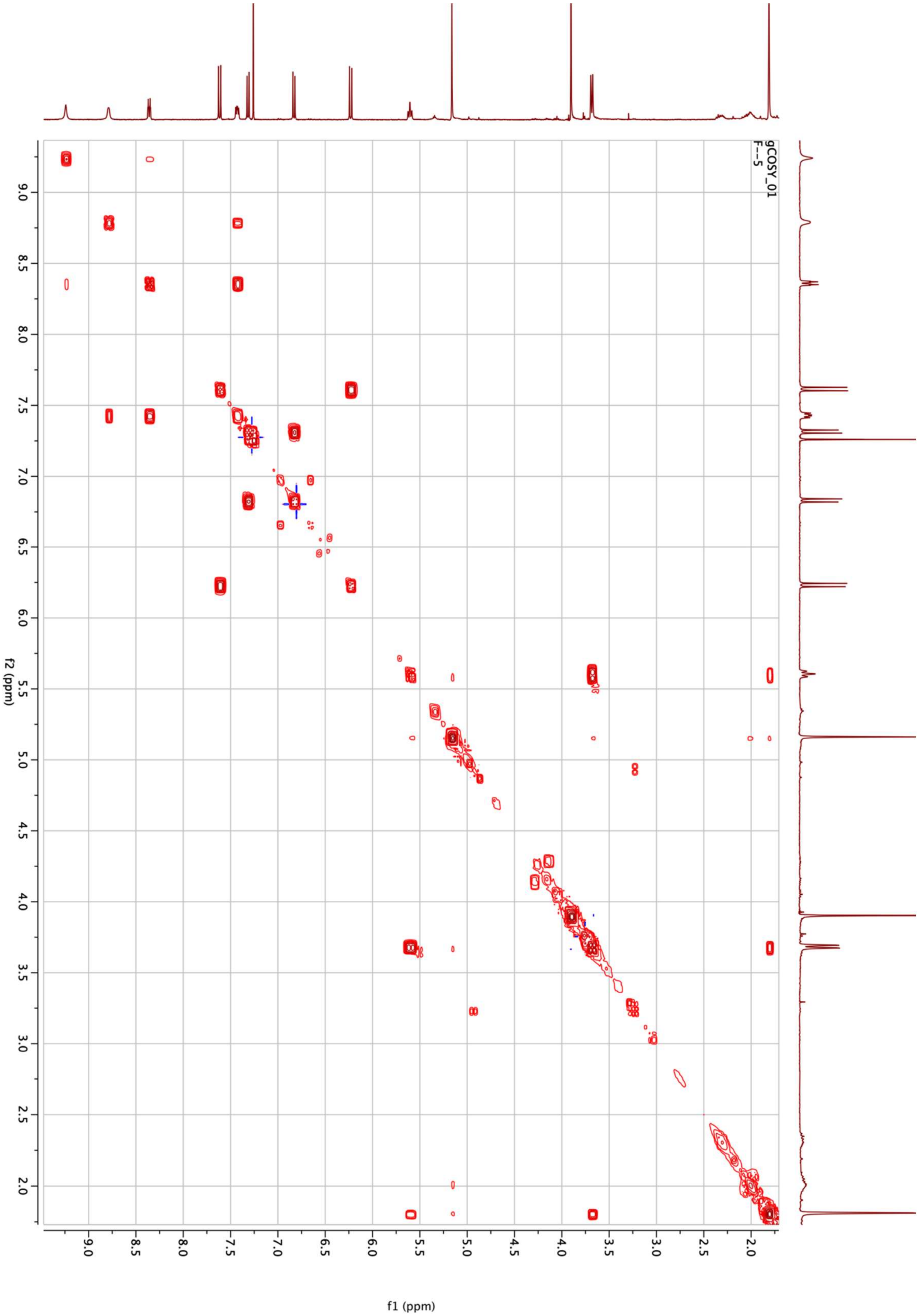

Fig. S18 2D COSY spectrum of Neopapillarine (**4**)

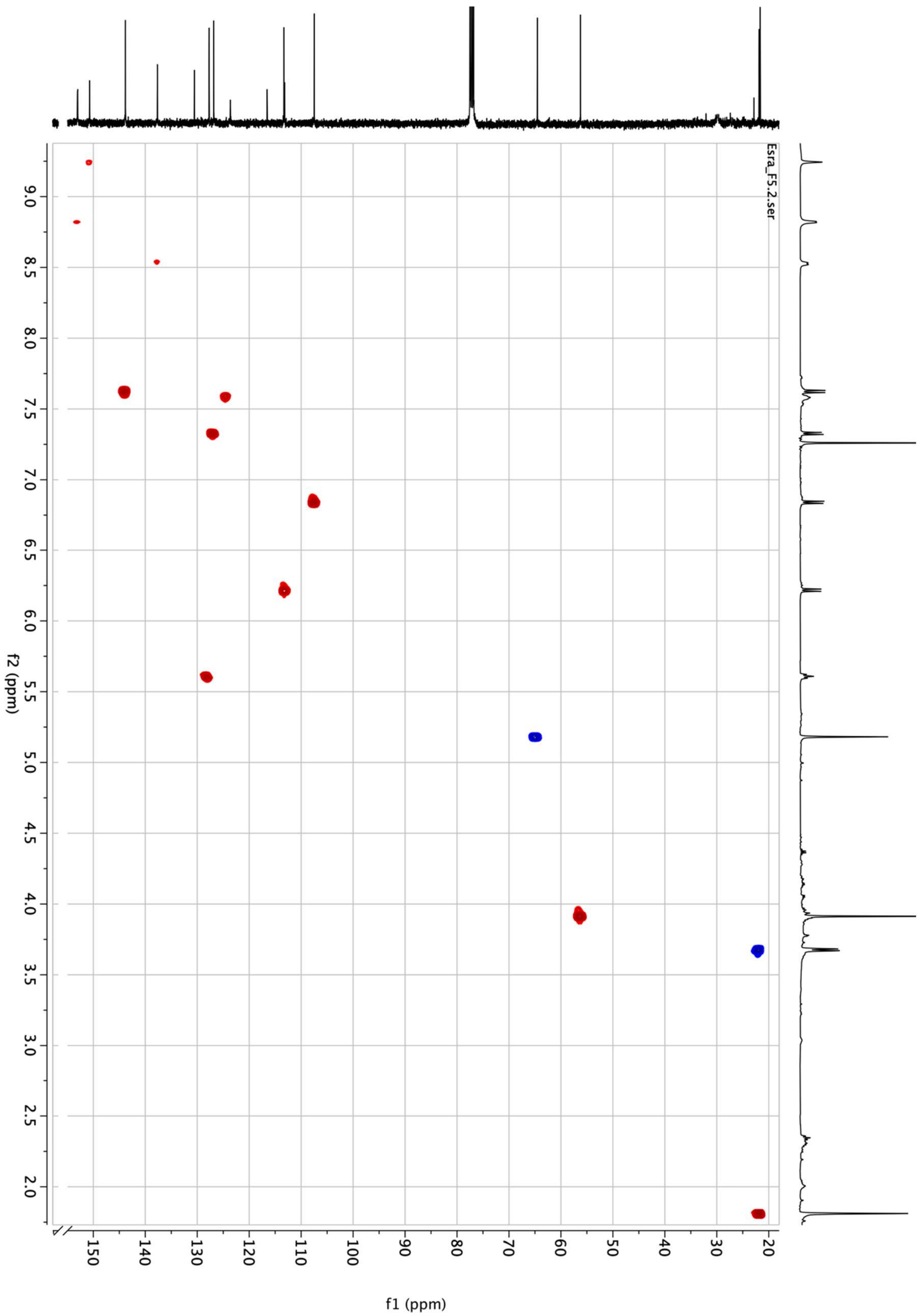

Fig. S19 2D HSQC spectrum Neopapillarine (**4**)

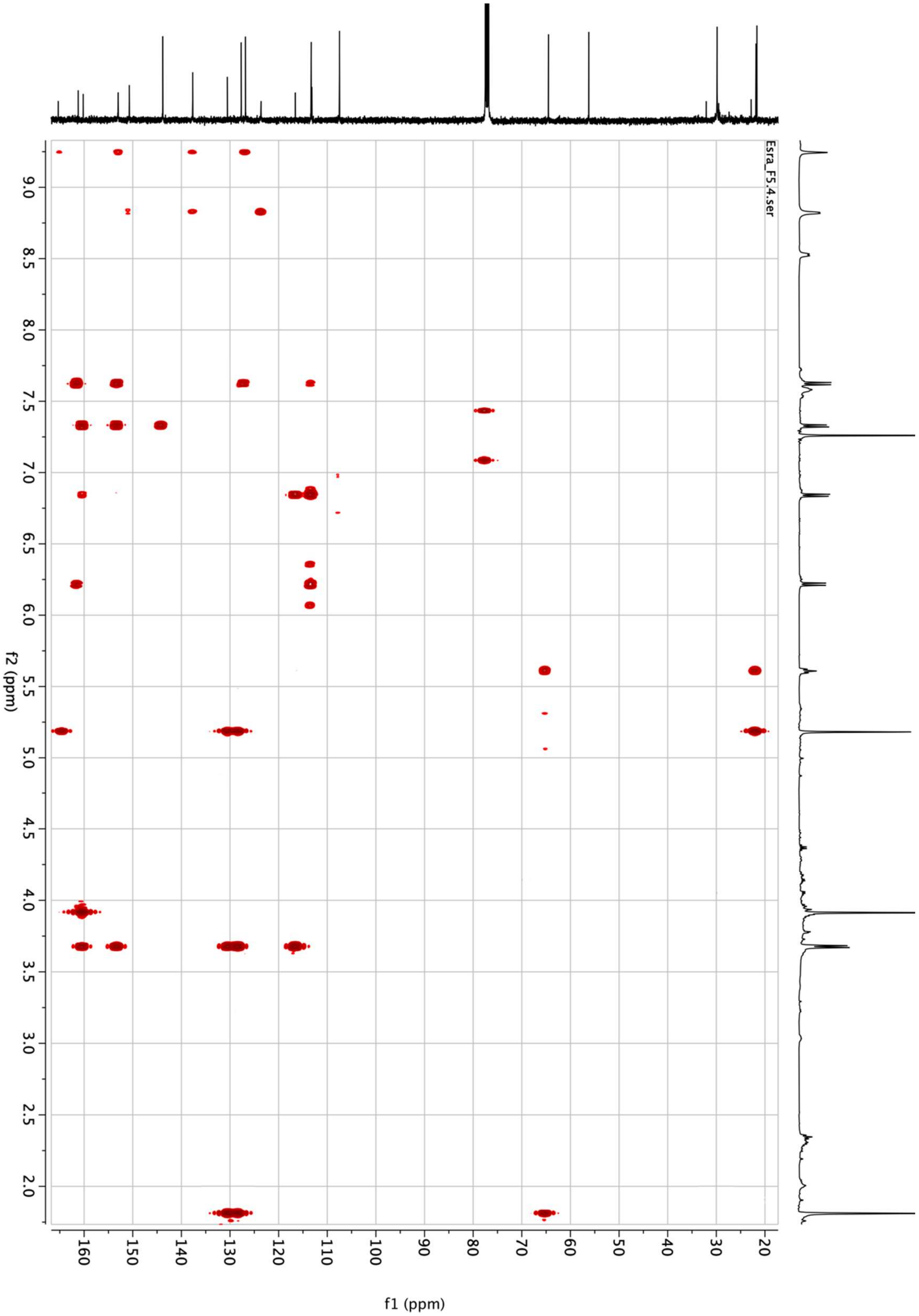

Fig. S20 2D HMBC spectrum of Neopapillarine (**4**)

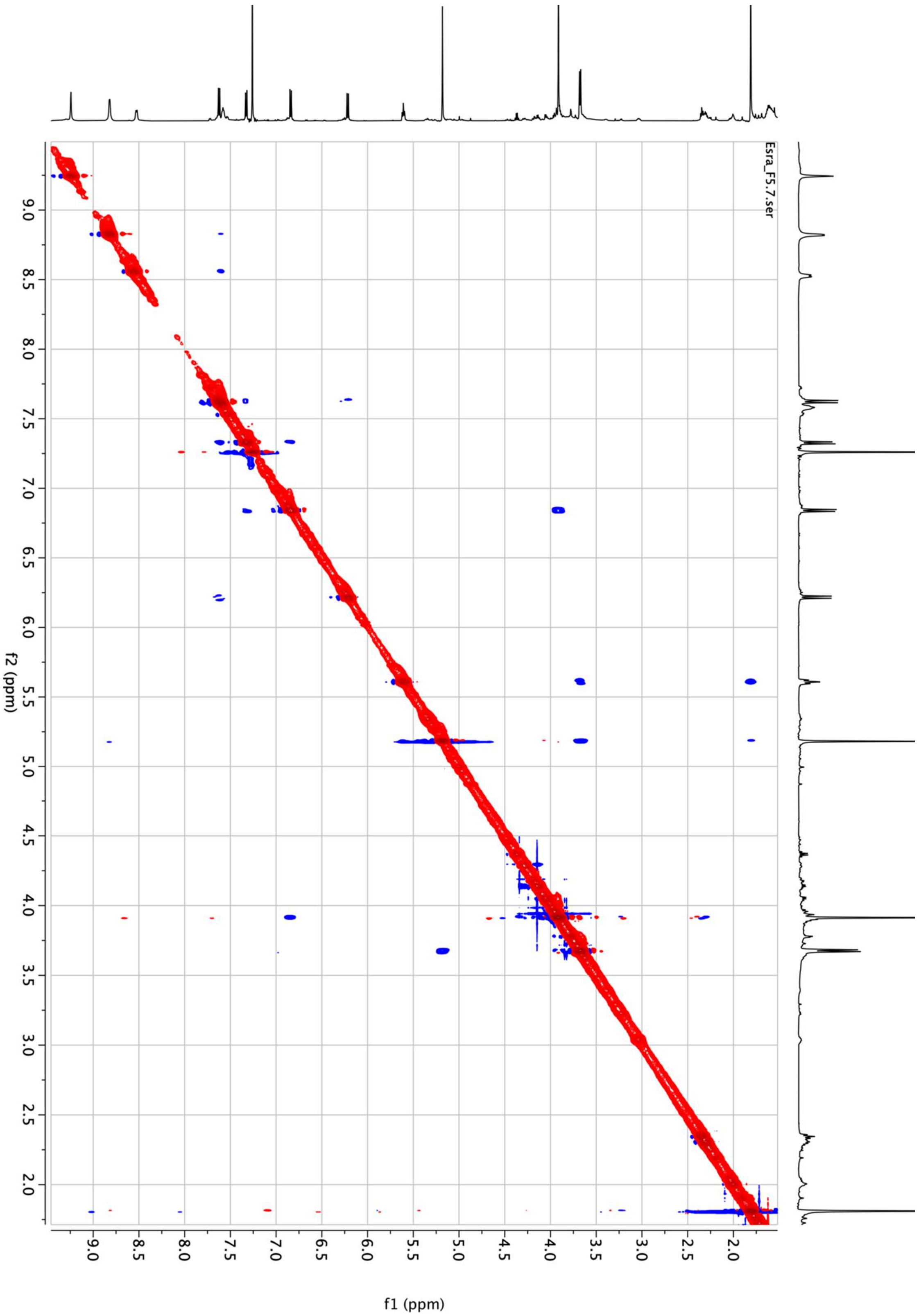

Fig. S21 2D NOESY spectrum of Neopapillarine (**4**)

Qualitative Analysis Report

**Data Filename**	QT01191604.d	**Sample Name**	101I069B
**Sample Type**	Sample	**Position**	Vial 2
**Instrument Name**	Instrument 1	**User Name**	
**Acq Method**	SMaccms_DESI(+)_Centroid.m	**Acquired Time**	2/13/2018 10:20:10 AM
**IRM Calibration Status**	[redacted]	**DA Method**	Default.m
**Comment**			

Sample Group **Info.**
Acquisition SW 6200 series TOF/6500 series
Version Q-TOF B.05.01 (B5125.1)

User Chromatograms

Fragmentor Voltage 150 **Collision Energy** 0 **Ionization Mode** ESI

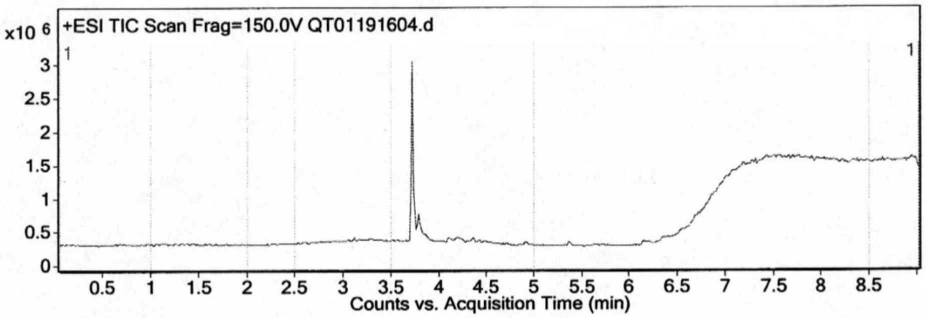

User Spectra

Fragmentor Voltage	Collision Energy	Ionization Mode
150	0	ESI

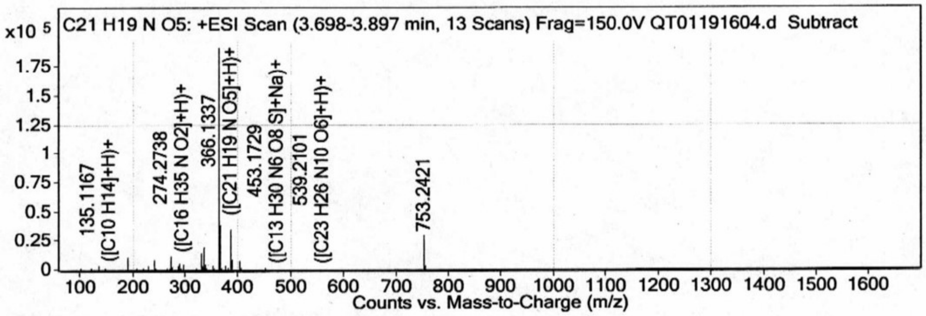

Peak List

m/z	z	Abund	Formula	Ion
191.1791	1	10605.98	C14 H22	(M+H)+
243.1013	1	7964.63	C15 H14 O3	(M+H)+
274.2738	1	10791.12	C16 H35 N O2	(M+H)+
332.2794	1	14030.35	C17 H35 N5	(M+Na)+
337.2347	1	18891.51	C18 H34 O4	(M+Na)+
366.1337	1	190952.17	C21 H19 N O5	(M+H)+
367.1367	1	38951.67	C21 H19 N O5	(M+H)+
388.1153	1	33976.99	C21 H19 N O5	(M+Na)+

Fig. S22 HRESIMS spectrum of Neopapillarine (**4**)

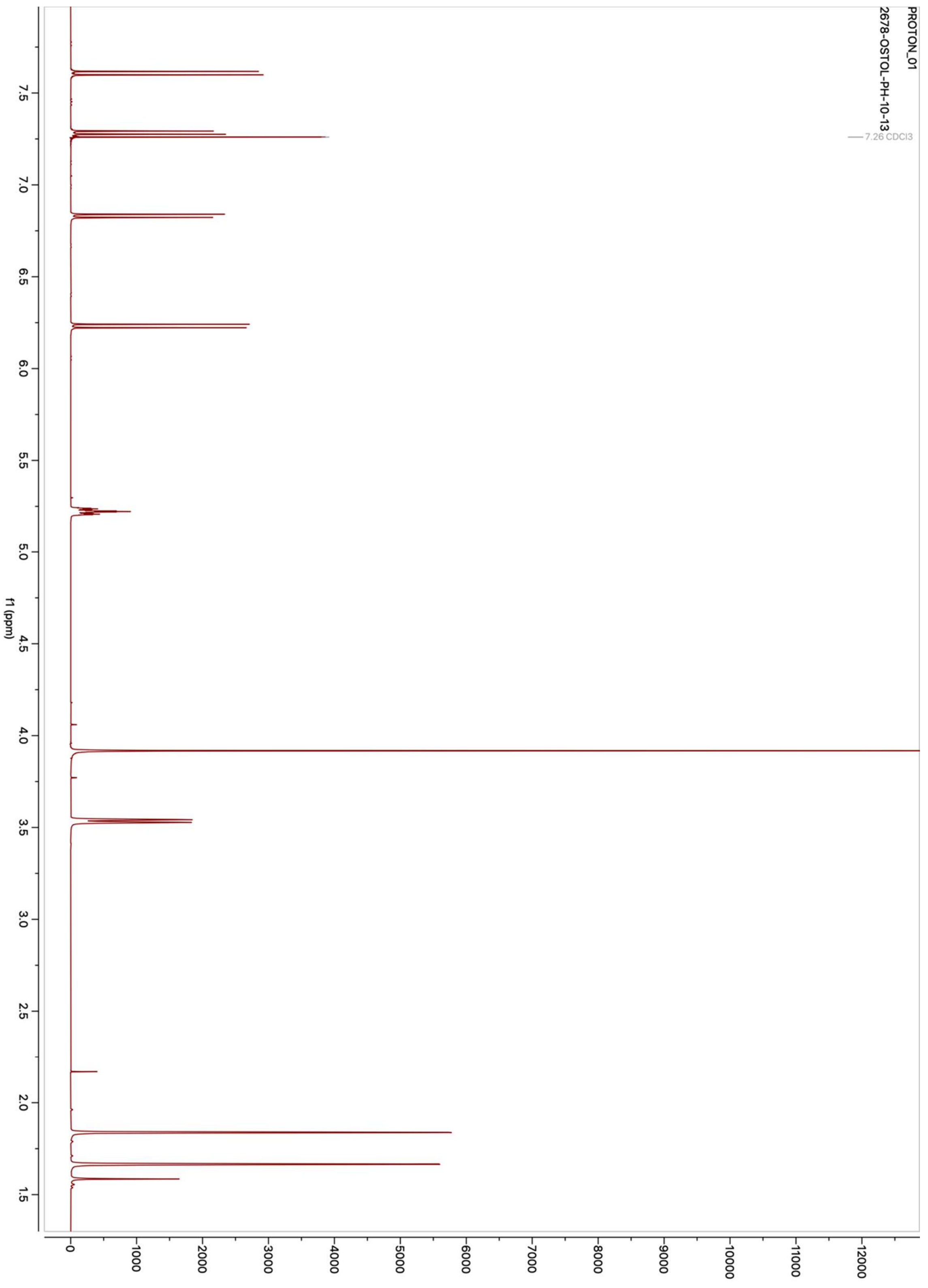

Fig. S23 ^{1}H NMR spectrum of Osthol (**1**)

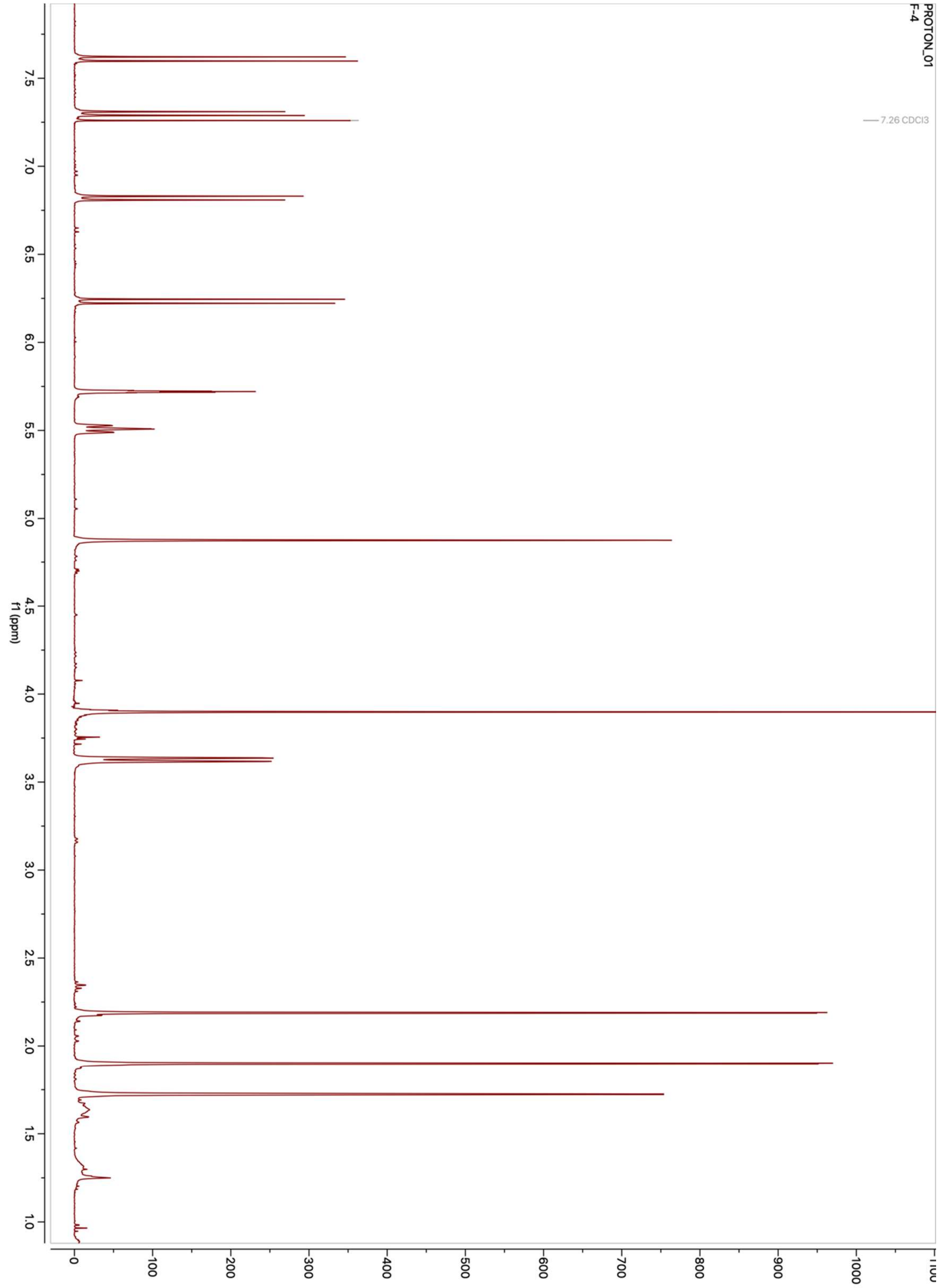

Fig. S24 ^{1}H NMR spectrum of 4′-Senecioyloxyosthol (**5**)

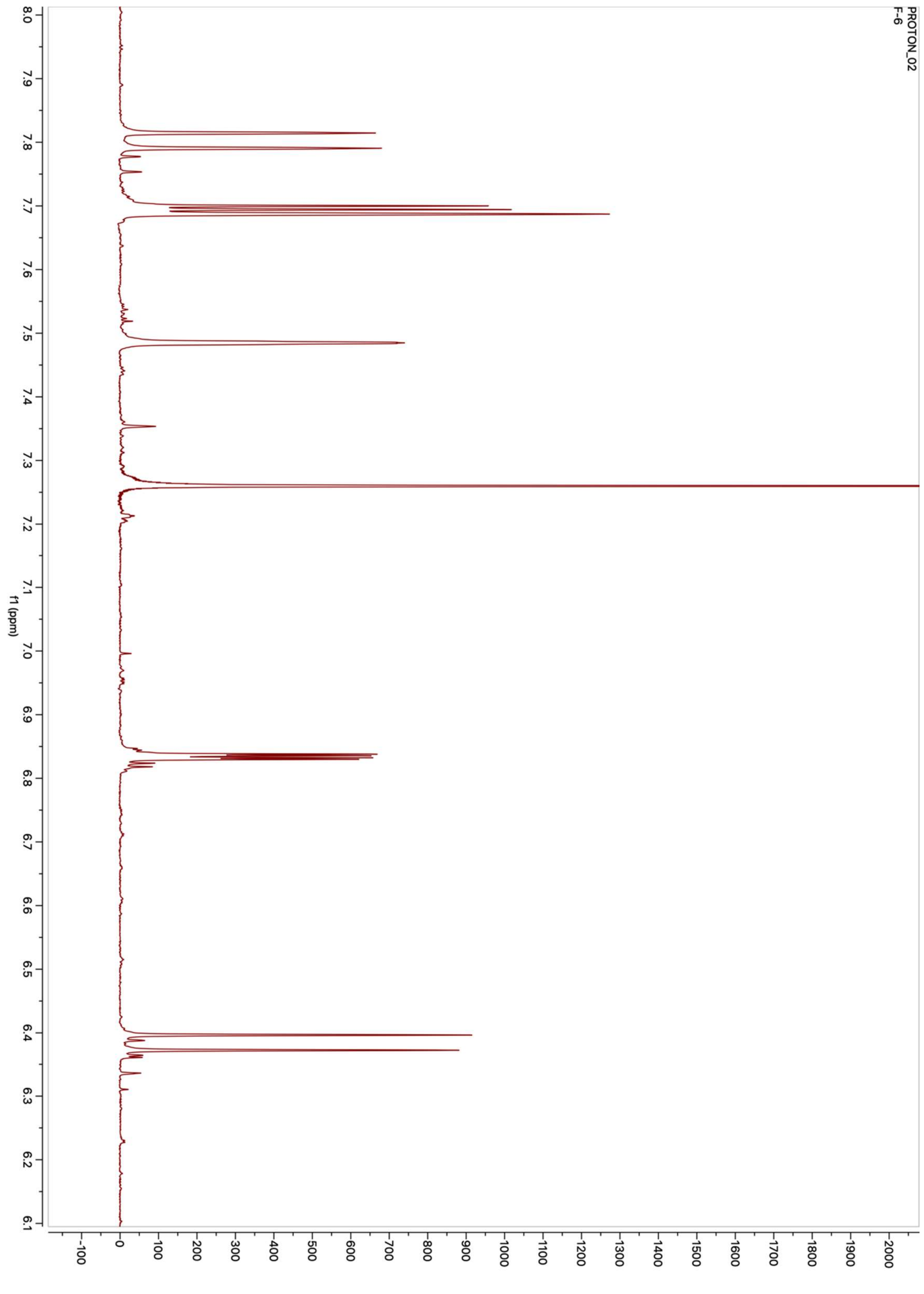

Fig. S25 Expanded ^{1}H NMR spectrum of Psoralen (**6**)

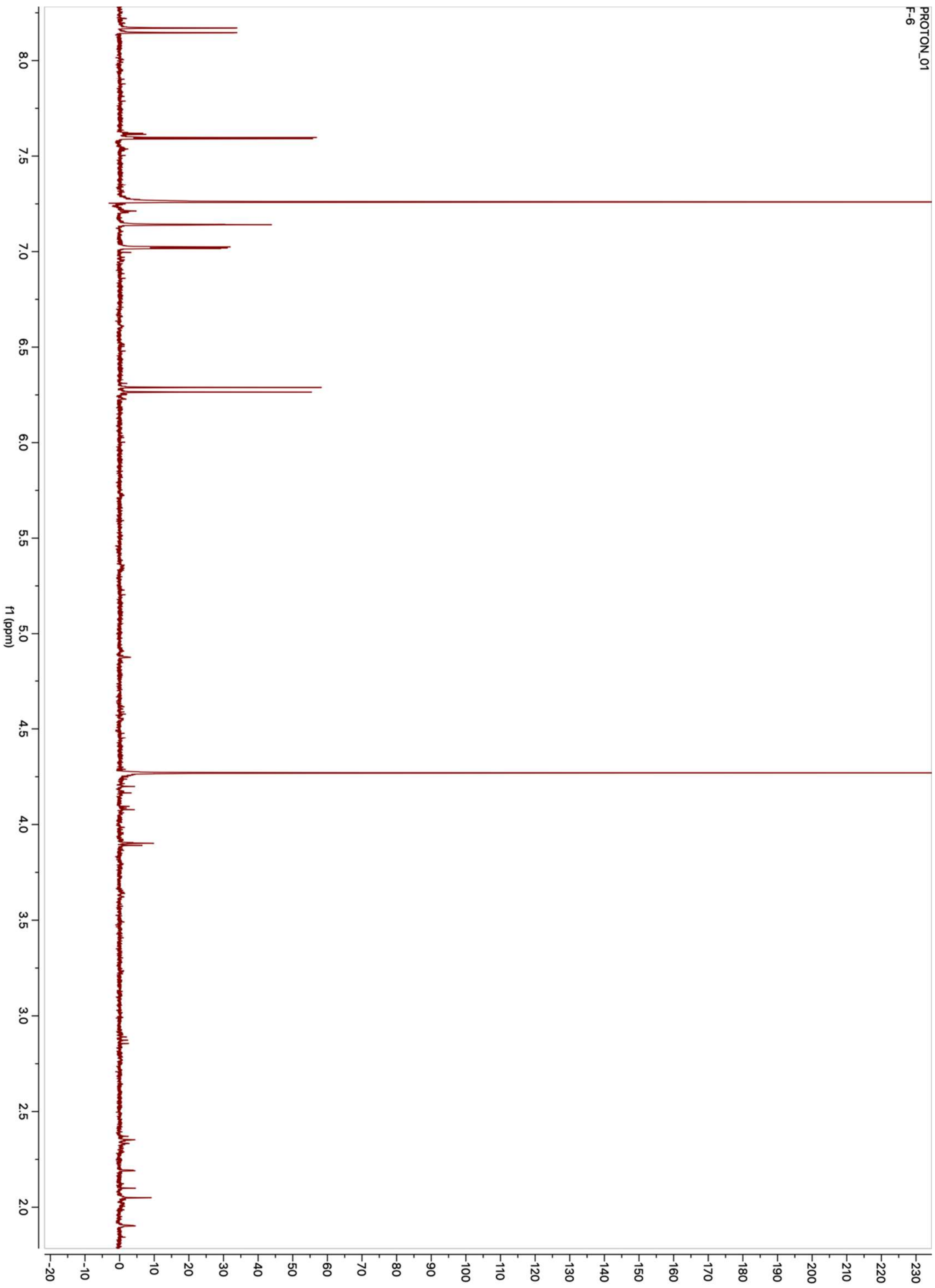

Fig. S26 ^{1}H NMR spectrum of Bergapten (**7**)

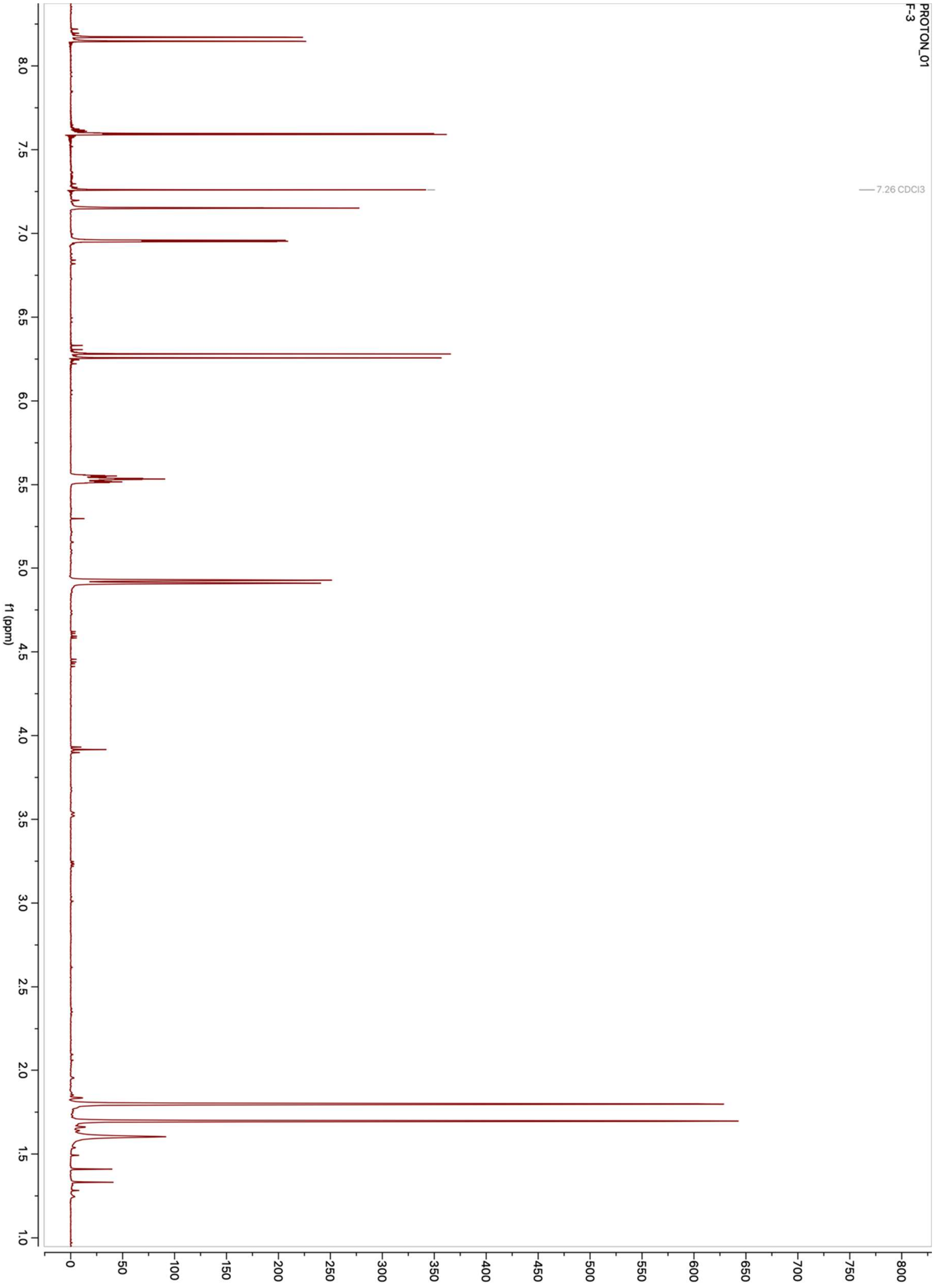

Fig. S27 ^{1}H NMR spectrum of Isoimperatorin (**8**)

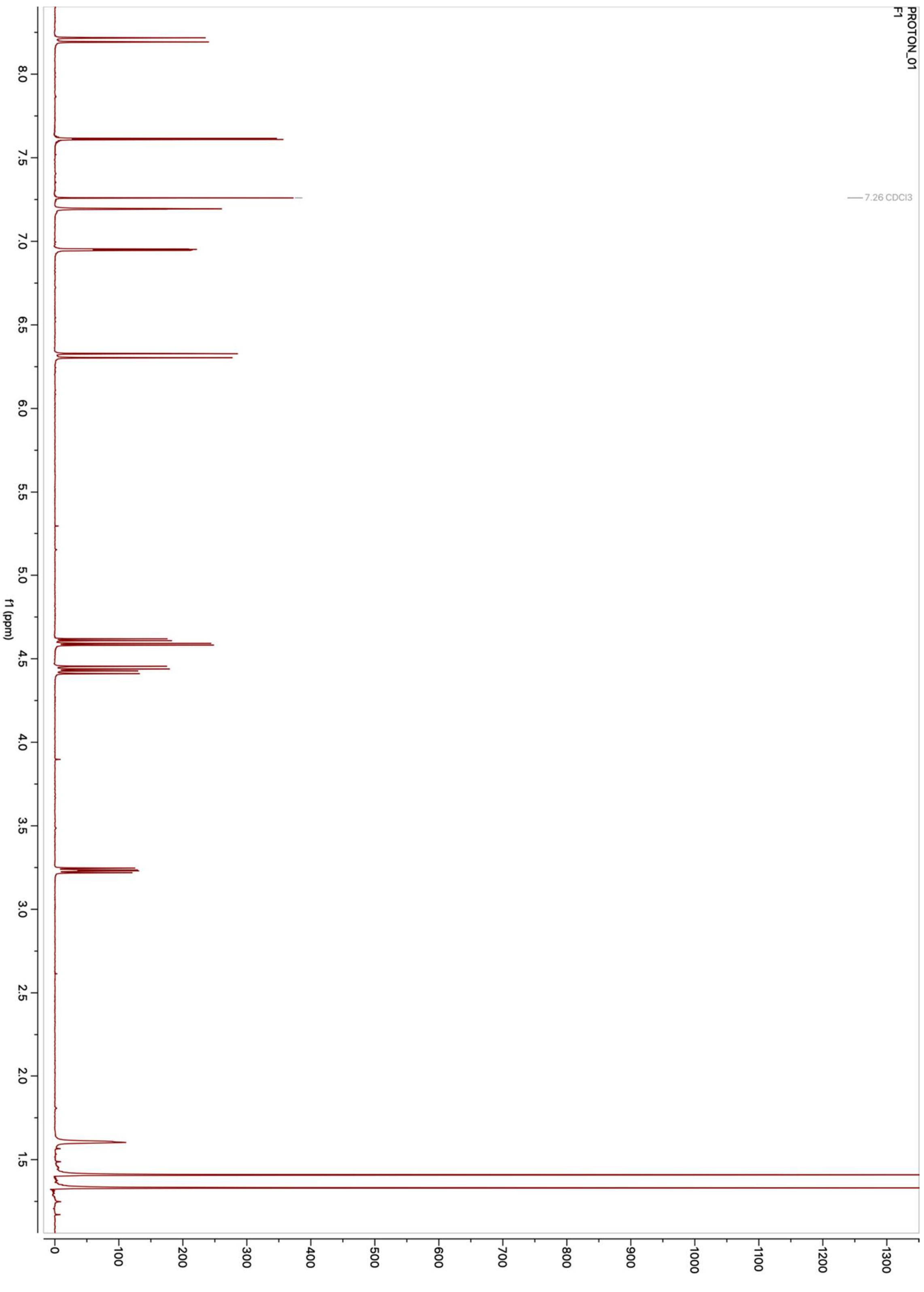

Fig. S28 ^{1}H NMR spectrum of Oxypeucedanin (**9**)

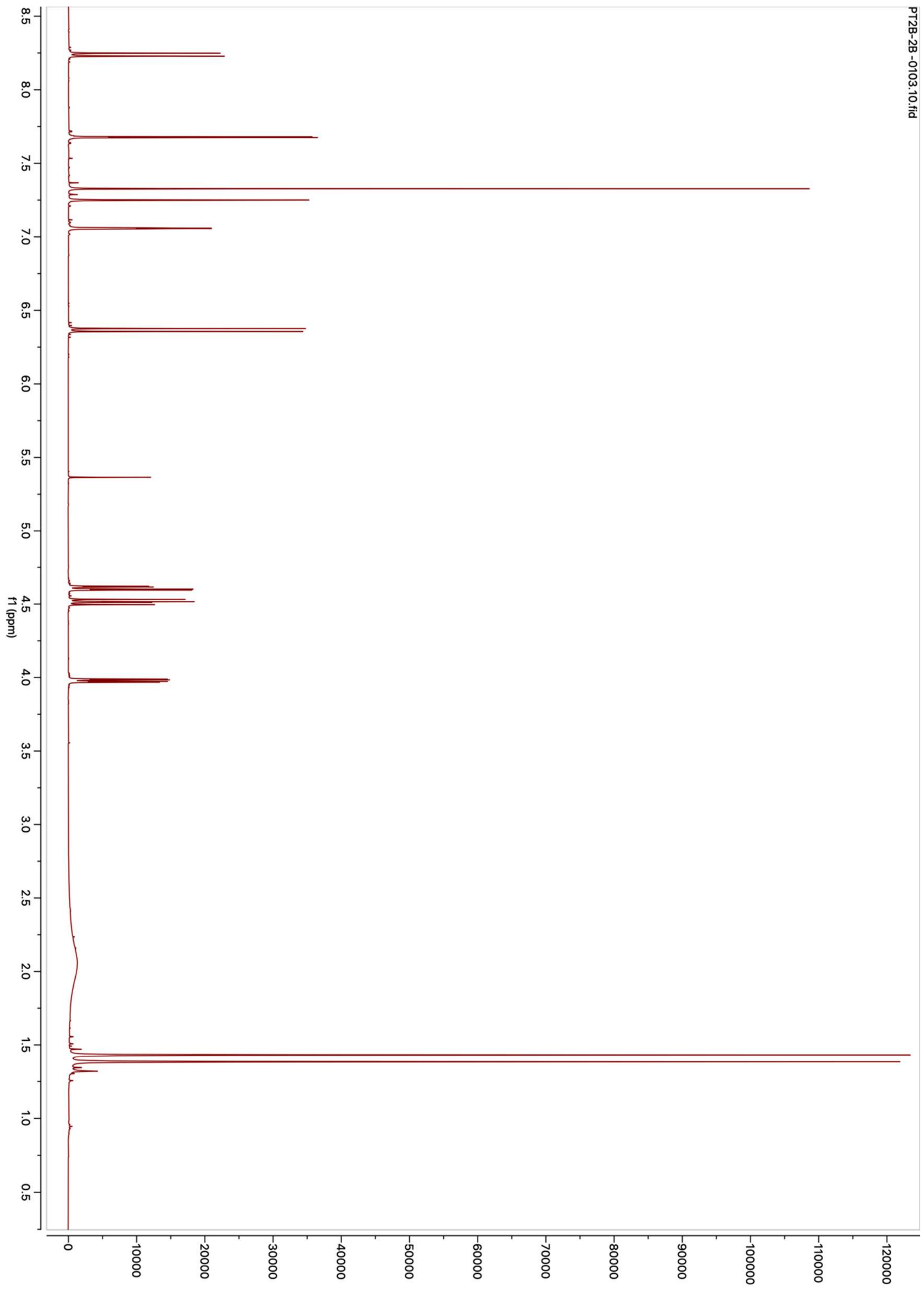

Fig. S29 ^{1}H NMR spectrum of Oxypeucedanin Hydrate (**10**)

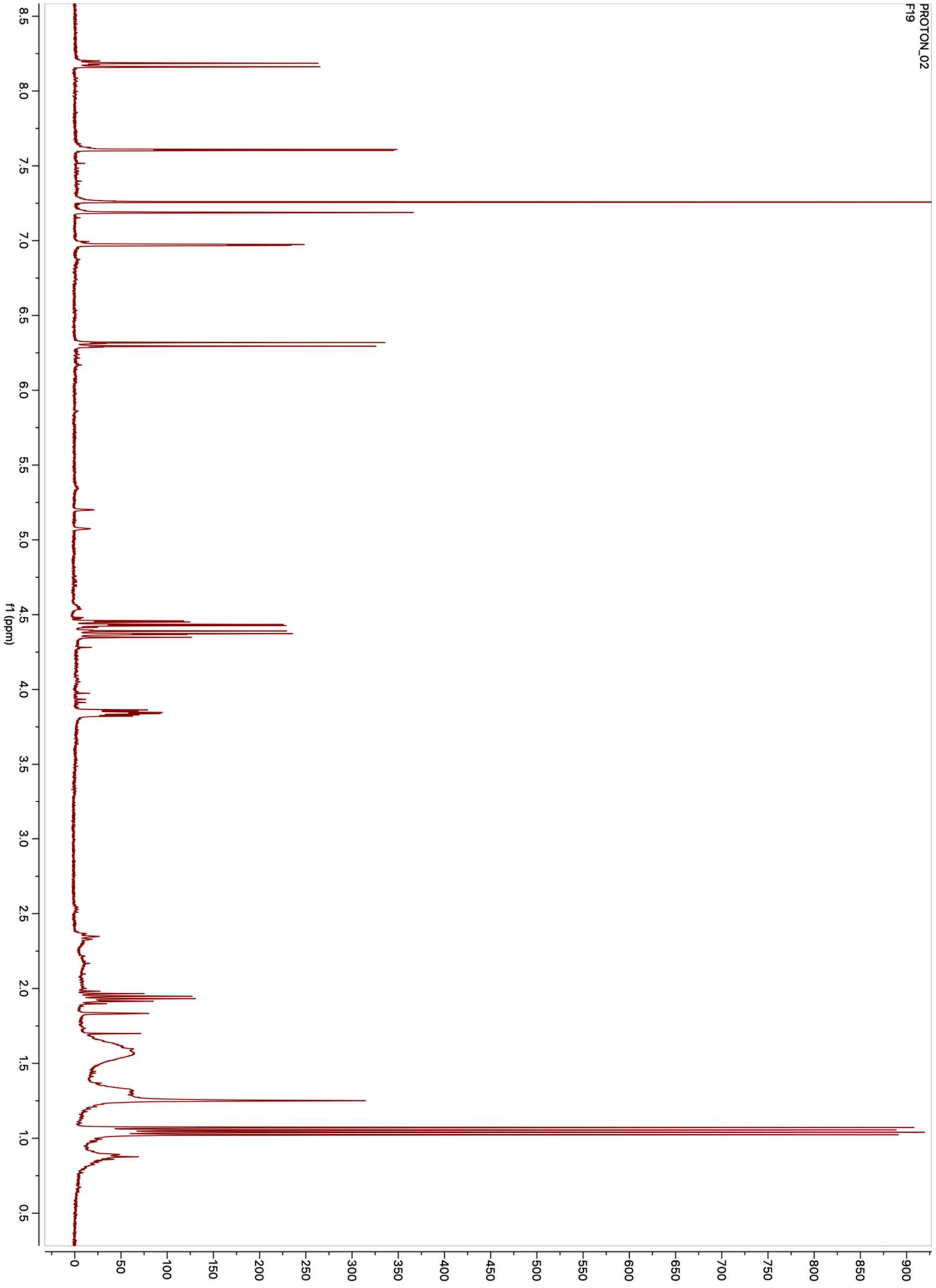

Fig. S30 ^{1}H NMR spectrum of Pranferol (**11**)

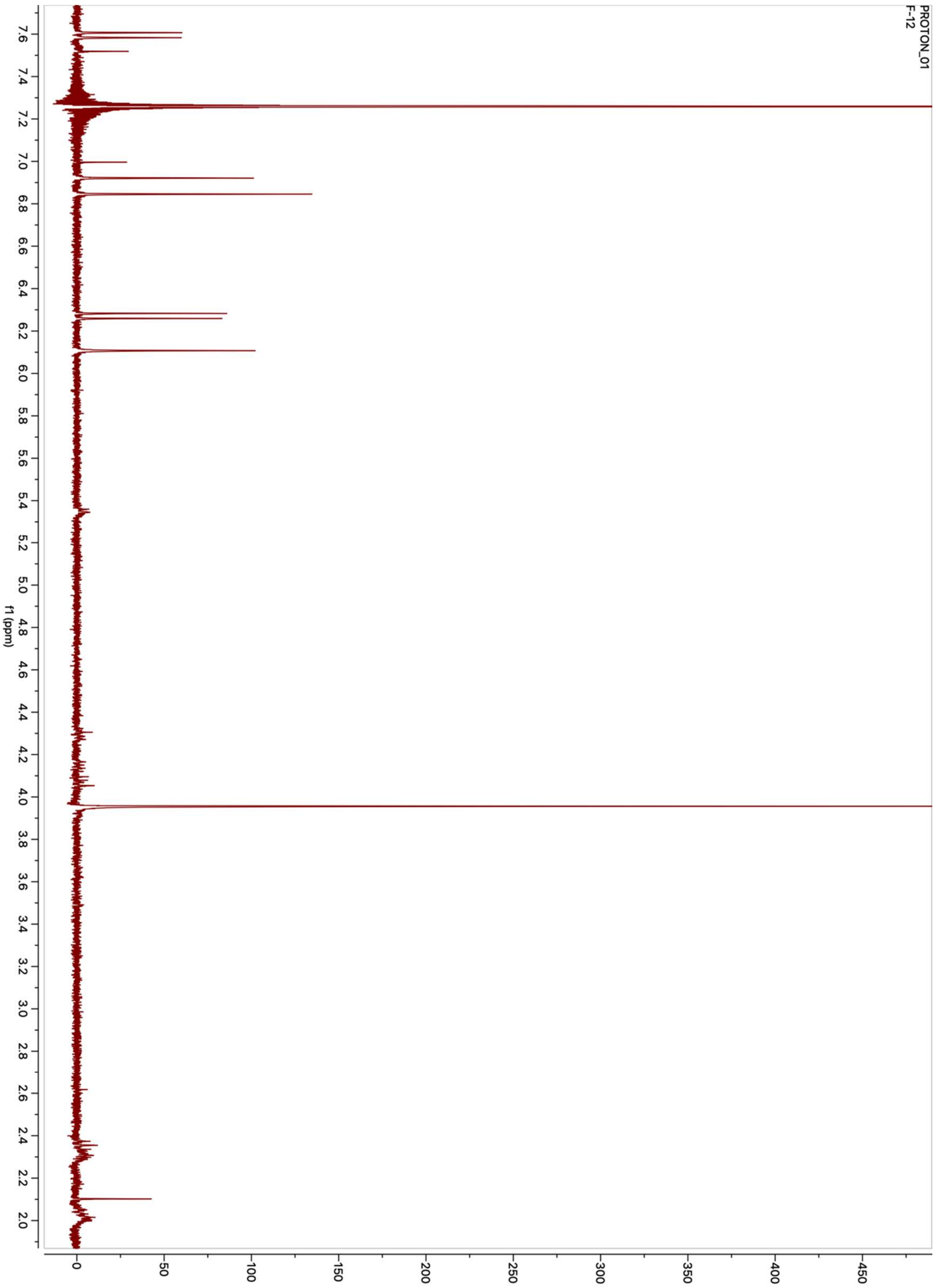

Fig. S31 ^{1}H NMR spectrum of Scopoletin (**12**)

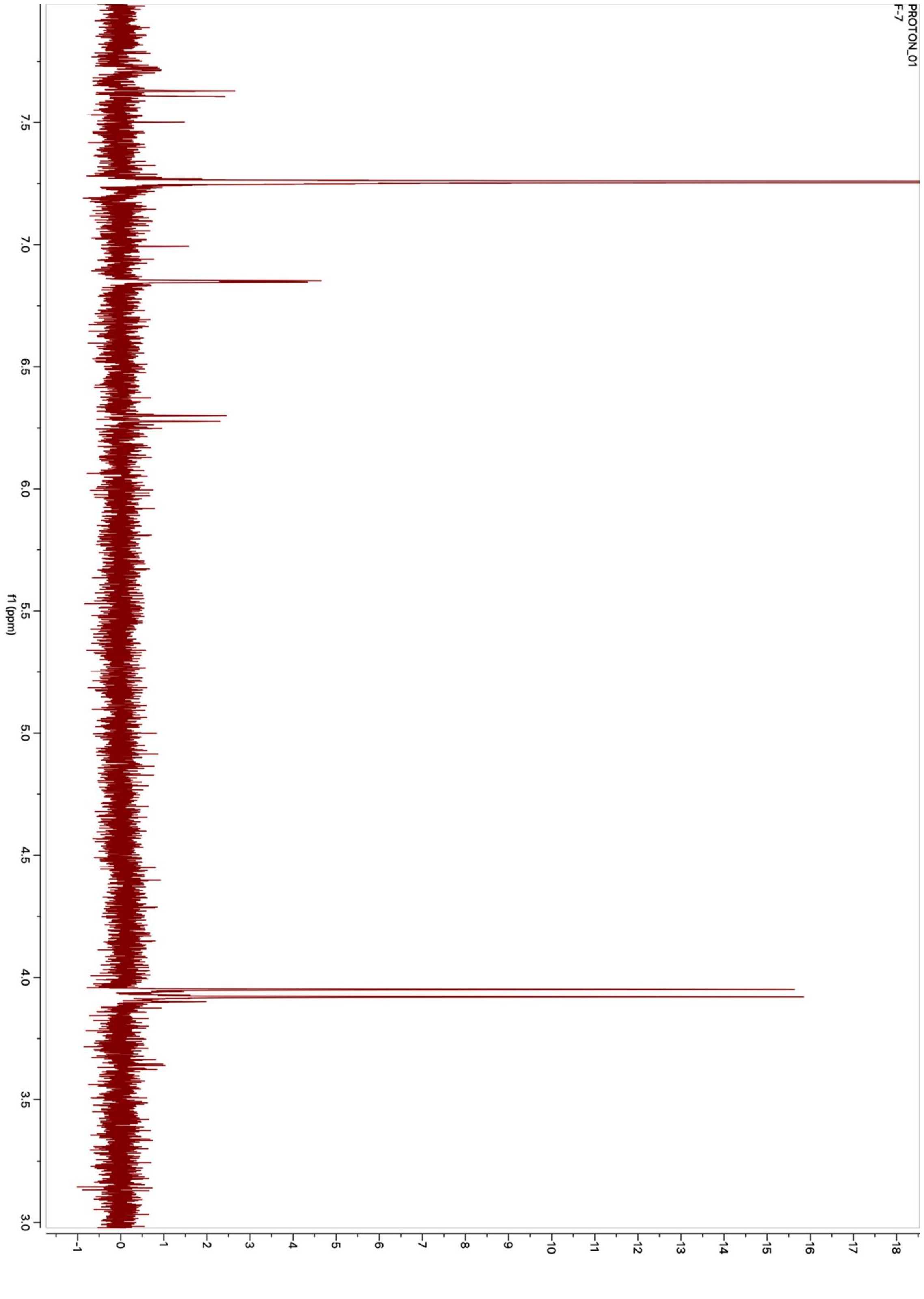

Fig. S32 ^{1}H NMR spectrum of Scoparone (**13**)
